# Functional Aromatic Polyamides

**DOI:** 10.3390/polym9090414

**Published:** 2017-09-05

**Authors:** José A. Reglero Ruiz, Miriam Trigo-López, Félix C. García, José M. García

**Affiliations:** Departamento de Química, Facultad de Ciencias, Universidad de Burgos, Plaza de Misael Bañuelos s/n, 09001 Burgos, Spain; jareglero@ubu.es (J.A.R.R.); mtrigo@ubu.es (M.T.-L.); fegarcia@ubu.es (F.C.G.)

**Keywords:** aromatic polyamides, aramids, high-performance polymers, functional polymers, advanced materials

## Abstract

We describe herein the state of the art following the last 8 years of research into aromatic polyamides, wholly aromatic polyamides or aramids. These polymers belong to the family of high performance materials because of their exceptional thermal and mechanical behavior. Commercially, they have been transformed into fibers mainly for production of advanced composites, paper, and cut and fire protective garments. Huge research efforts have been carried out to take advantage of the mentioned characteristics in advanced fields related to transport applications, optically active materials, electroactive materials, smart materials, or materials with even better mechanical and thermal behavior.

## 1. Introduction

Aromatic polyamides, wholly aromatic polyamides, or the shorter form aramids, stand for synthetic polyamides comprised >85% by amide groups (–CO–NH–) bound directly to two aromatic rings [1]. These polymers are categorized as high-performance materials because of their outstanding mechanical strength and superior high thermal resistance. They are spun into fibers for advanced fabrics such as advanced sport and work protective clothing, bullet-proof body armor, advanced composites in armament and aerospace industries, in composites as asbestos substitutes, high-temperature insulation paper, to name but a few [2].

The structure of the aramids is based on the rigid aromatic amide linkage, which is responsible for the outstanding properties of these materials. Highly directional and efficient interchain hydrogen bonds are established, giving rise to materials with high crystallization tendency and extremely high cohesive energy density. At the same time, they are also responsible of the insolubility of the wholly aromatic polyamides, a drawback that impair the expansion of the application field of these materials, where the improvement of the solubility is a topic of research interest.

From a historical viewpoint, poly(*p*-benzamide) was commercialized about 50 years ago, for a short period of time, under the tradename ‘Fiber B^®^’. It was an all-*para* oriented aramid, an extremely rigid rod-like macromolecule, with important transformation impairments, that was replaced on the market in the 1970 decade by poly(*p*-phenylene terephthalamide) (PPTA), the unique all-*para* oriented aramid commercialized nowadays. On the other hand, in 1967 was marketed poly(*m*-phenylene isophthalamide) (MPIA), a less rigid and easily-transformable aramid with impressive thermal resistance, only slightly underperforming the mechanical behavior of PPTA. PPTA and MPIA were discovered and initially commercialized under the tradenames of ‘Kevlar^®^’ and ‘Nomex^®^’ by Dupont. The year 1987 saw the commercialization of the third aramid marketed today, a copolymer with enhanced solubility prepared with terephthaloyl dichloride (TPC) and *p*-phenylenediamine (PPD), like PPTA, and also with a diamine based on diaminodiphenyl ether having *m*- and *p*-oriented amine groups (3,4′-diaminodiphenyl ether (ODA)), giving rise to an aramid with both *m*- and *p*-oriented aromatic rings (ODA/PPTA). It was put on the market by Teijin with the tradename “Technora^®^”. The structures and some of the aramid producers are shown in Table 1.

The exploitation of the above-mentioned characteristics of aramids is a topic of current interest. Thus, aromatic polyamides are currently being studied as functional and cutting-edge materials for even better thermally and mechanically resistant polymers, sensing and extraction applications, films and membranes for transport applications, optically active materials, materials with electromechanical or electrochromic properties, and functional aramids with selected chemical groups and lateral structures. Accordingly, this review focuses on recent advances, from 2009, in design, preparation, and potential applications of these materials. Previous developments in the field were summarized by Marchildon and by us in previously published reviews [2,3].

## 2. Synthesis and Processing

### 2.1. Low and High Temperature Solution Synthetic Methods

Aramids are synthesized at a lab scale by two procedures: low and high temperature solution methods. The commercial aramids PPTA and MPIA are prepared following the low temperature solution method from PPD and TPC, or *m*-phenylenediamine (MPD) and isophthaloyl dichloride (IPC), using a polar aprotic solvent, such as *N*-methyl-2-pyrrolidone (NMP) or *N*,*N*-dimethylacetamide (DMA), and salts as solubility promoters, such as LiCl and/or CaCl_2_. Aromatic diacids can be used, instead of the moisture sensitivity diacid chlorides, using the high temperature solution method, described by Yamazaki et al. [4]. These methods and the procedures followed for the commercial preparation of aramid fibers are reported in our previous reviews and book chapters [2,5,6].

### 2.2. Alternative Synthetic Methods

From the organic chemistry viewpoint, the aromatic amide group can be prepared by a number of alternative reactions to prepare wholly aromatic polyamides [2,7,8,9,10]. However, only the previously described low and high temperature solutions methods have both demonstrated commercial and laboratory viability in relation with the absence of side reaction and the quantitative yield required for obtaining high molecular mass polymers. Nevertheless, among the time span covered by this review and at lab scale, alternative procedures to the conventional condensation of diacid or diacid dichloride and diamines have been reported. From the green chemistry viewpoint, Ueda et al. reported on the solid-state preparation of aramids [11,12], and Kobashi et al. discussed the direct condensation of aromatic diacids and diamines at high temperature without condensation promoters [13].

### 2.3. Processing

Commercial aromatic polyamides are processed into staple fibers, continuous multifilament yarn, staple fibers and pulp from solution by wet spinning (MPIA, ODA/PPTA and PPTA), dry spinning (MPIA and ODA/PPTA) and dry-jet wet spinning (MPIA and ODA/PPTA) [6].

## 3. Synthesis of Functional Aromatic Polyamides with Controlled Structure

For the sake of simplicity, it is generally assumed that all the functional groups of the monomers and growing polymer chains present same or similar reactivity. Under this assumption, conventional polycondensation reactions occur on a step-growth manner according to Carother’s equation and Flory’s statistical description [14]. The control over the molecular mass and the polymer end groups is difficult, and thus the molecular mass distribution approaches a value of 2 at high conversion. However, when monomers with evident unequal reactivity are employed, or when a change of reactivity is induced in one functional group by reaction and bond formation during polymerization of other functional group, the polymer growth do not obey these conventional rules. In order to synthesize a polymer with controlled molecular mass, a narrow molecular mass distribution and defined chain-end functional groups, the condensation should proceed in a chain-growth polymerization manner instead of step-growth polycondensation. This is the case for so-called living polymerization.

### 3.1. Chain-Growth Polycondensation

**Scheme 1:**
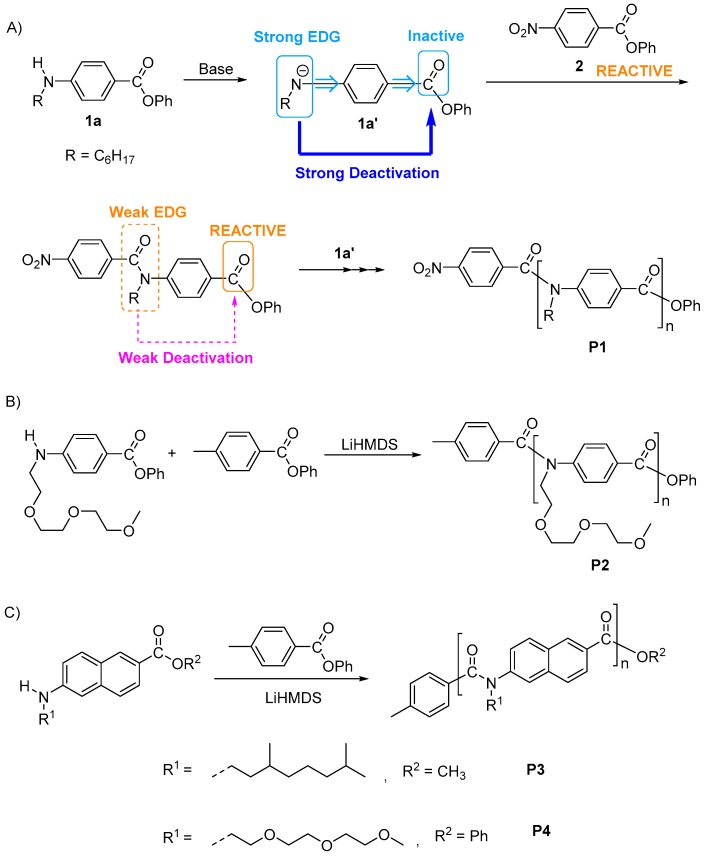
Aromatic polyamides with controlled polydispersity and mass obtained by chain-growth polycondensation. (**A**) polycondensation of phenyl 4-(octylamino)benzoate and the initiator phenyl 4-nitrobenzoate; (**B**) polycondensation of a monomer that bears a TEG sidechain; (**C**) polycondensation o 2,6-positions substituted naphthalenes.

Some experimental results showed that it is possible to obtain high molecular mass condensation polymers even at low conversions, contrary to Flory’s and Carother’s predictions. This opened the way to chain-growth polycondensation. Yokoyama and Yokozawa published a detailed review of chain-growth polycondensation [15,16], and a more recent review about the latest developments in the field [17]. Polyethers and aromatic polyamides are prepared using the unequal reactivity induced by activation of the end groups of the polymer chains. They found that the polycondensation of phenyl 4-(octylamino)benzoate 1a (Scheme 1A, P1) and the initiator phenyl 4-nitrobenzoate 2 in the presence of a base yielded well defined aramids with low polydispersity (*M*_w_/*M*_n_ ≤ 1.1, where *M*_n_ and *M*_w_ are number-average and weight-average molecular masses, respectively). The fact that the *M*_n_ values increase with the monomer proportion can be attributed to the difference in substituent effect between the monomer and the polymer, indicating a chain-growth polycondensation reaction. The amide anion 1a′, which is formally an AB condensation monomer, do not polymerize because of the strong electron-donating ability (electron-donating group—EDG) of the amide anion. On the contrary, the electron-withdrawing group (EWG) nitro group activates the phenyl ester moiety in the initiator 2 through resonance effects. The reaction of 1a′ with 2 yields an amide, which is a weak EDG, and so the monomer 1a′ reacts only with the more reactive phenyl ester of the amide of the growing polymer chain, and not with itself. Therefore, the polymerization follows a chain-growth pattern. Good results were obtained by using a strong base, such as lithium hexamethyldisilazide (LiHMDS), and phenyl 4-methylbenzoate as the initiator [18]. However, when the monomer bears a tri(ethylene glycol) monomethyl ether (TEG) side chain instead of an alkyl group, the polymerization is very slow as a result of the coordination of the Li cation to the nucleophilic nitrogen atom of the side chain and the concomitant reduction of the reactivity of the amide. The polymerization of the methyl ester monomer gave a polymer with low molecular mass and broad polydispersity. On the other hand, the more reactive phenyl ester monomer yielded at −20 °C a polymer with controlled structure, i.e., controlled molecular mass and low polydispersity (Scheme 1B, P2) [19].

The resonance effects also work in the 1,5- or 2,6-positions of naphthalene, so Mikami et al. extended their work using this type of monomers (Scheme 1C, P3–P4) [20]. Well defined polymers were obtained using the monomer with a 3,7-dimethyloctyl pendant group, whereas the monomer with the TEG moiety gave both chain-growth and step-growth polyamides, with self-condensation, because an insufficient deactivation of the electrophilic ester moiety, rendering polymers with *M*_n_ lower than the expected theoretical value.

**Scheme 2:**
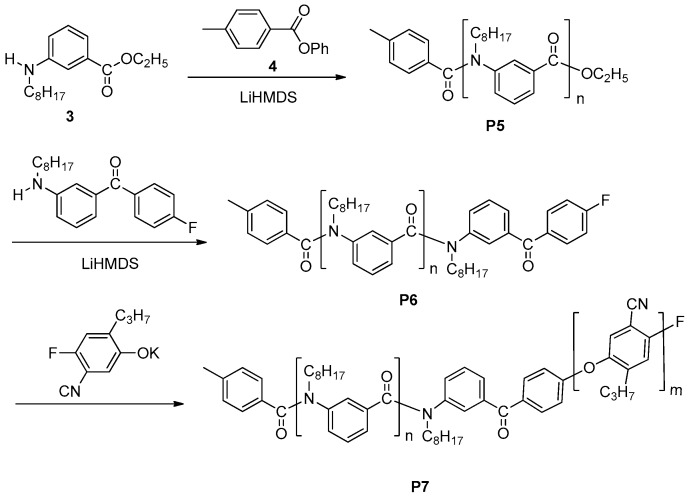
Block polymers obtained by chain-growth polycondensation.

Following this methodology, block copolymers were obtained exploiting the living-like polycondensation. Yokoyama et al. [21] reported a controlled synthesis of a diblock copolymer composed of an aromatic polyamide and an aromatic polyether, consisting of *N*-alkylated poly(*m*-benzamide) and cyano and propyl substituted poly(1,4-phenylene oxide), by two successive chain-growth condensation polymerizations (Scheme 2, P5–P7). The synthetic route started with the polymerization of 3 from a monofunctional initiator, followed with the introduction of a fluorobenzophenone unit at the polymer terminal, and finished with the polymerization of the polyether monomer.

**Scheme 3:**
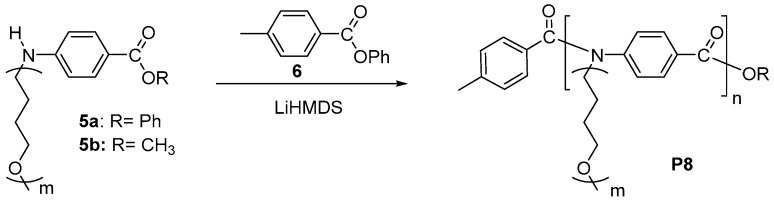
Well-defined aramid grafted with poly(tetrahydrofuran).

Regarding graft copolymers, Sugi et al. described a well-defined aramid grafted with poly(tetrahydrofuran) by chain growth polymerization of 4-aminobenzoic acid ester having a poly(tetrahydrofuran) residue 5a on the amino group [22]. The polymerization of monomer 5a at −10 °C was accompanied with self-polycondensation. However, polymerization of the methyl ester monomer 5b at 10 °C occurred only in a chain growth manner to render a well-defined graft copolymer P8 with a narrow molecular mass distribution (Scheme 3, P8).

**Scheme 4:**
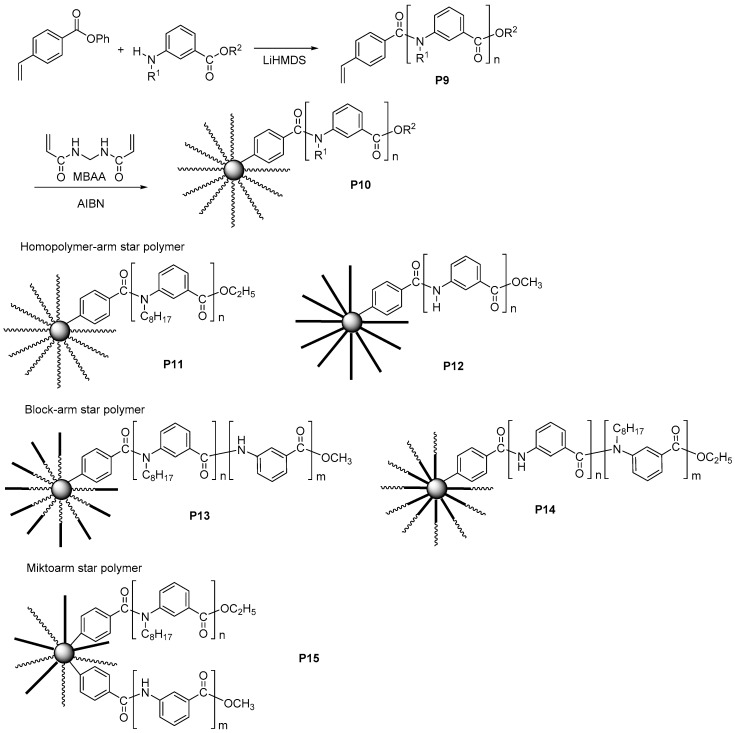
Star polymers prepared by chain-growth polycondensation/radical copolymerization.

Self-assembly of star polymers would also be a useful starting point to prepare nanoarchitecture of aramids, due to their compact structure, globular shape, and large surface area [23]. Ohisi et al. synthesized a star polymer with controlled molecular mass and low polydispersity following a chain growth condensation strategy using 3-(alkylamino)-benzoic acid esters (condensation monomer) in the presence of phenyl 4-vinylbenzoate (condensation initiator, vinylic co-monomer) and *N*,*N*′-methylenebisacrylamide (divinyl co-monomer) (Scheme 4, P9–P15) [24].

### 3.2. Constitutional Isomerism

The different reactivity of condensation monomers arises from asymmetry (asymmetry of monomers or induced by reaction of one functional group). Asymmetry is due to chemically inequivalent functional groups on the monomer that exhibit different reaction rates or kinetics.

The study of the polyamides obtained from asymmetric diamines or diacid monomers (XabX) has not been analyzed deeply. Polyamides based on asymmetrically substituted diamine monomers present unequal reactivity of functional groups, affecting thermal and mechanical properties, as described by Hsiao et al. [25]. Also, a good description of the relation between constitutional isomerism and physical properties can be found in the work of Pino et al. [26]. This work indicated that the quantification of the structure regularity is defined using the probability of finding two-adjacent non-symmetric units in a chain pointing in the same direction (probability parameter, *s*). Then, a polycondensation reaction of two monomers, symmetric and non-symmetric, can produce an infinite number of disordered polymers, in which *s* varies between 0 and 1. The authors also describe two limited ordered structures head to tail (H-T, *s* = 1) and head to head/tail to tail (H-H/T-T, *s* = 0).

The reaction between XabX and YccY produces short sub-structures –acca–, –accb–, –bcca– and –bccb– (–accb– and –bcca– are indistinguishable). Equation (1) describes the calculation of the probability parameter *s*, of –accb– sub-structures, related to the other three possible structures.

(1) $$s=\frac{\left[\mathrm{accb}\right]}{\left(\left[\mathrm{acca}\right]+\left[\mathrm{accb}\right]+\left[\mathrm{bccb}\right]\right)}$$

**Scheme 5:**
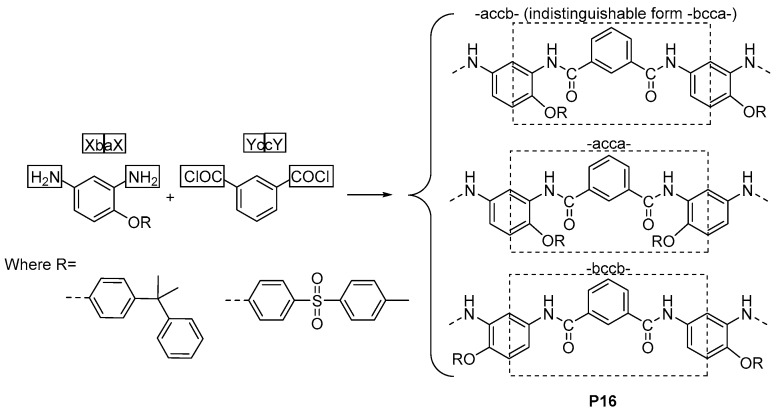
Aramid sub-structures obtained using an asymmetric AB monomer.

Recently, Pal et al. [27] studied the constitutional isomerism of aromatic polyamides containing pendant groups based on asymmetrically substituted *m*-phenylene diamines (Scheme 5, P16), where the sequence distribution can be analyzed and predicted. The polyamides were synthesized by low temperature interfacial polycondensation of 4-[4-(1-methyl-1-phenylethyl)phenoxy]-1,3-diamino benzene and 4-{4-[(4-methylphenyl)sulphonyl]phenoxy}-1,3-diamino benzene with two aromatic diacid chlorides, isophthaloyl chloride and terephthaloyl chloride. They obtained medium molecular mass, essentially amorphous polymers, with glass-transition temperatures in the range 237–254 °C, good thermal stability, and improved solubility. The constitutional isomerism was investigated by ^1^H and ^13^C nuclear magnetic resonance spectroscopy, finding a constitutional order *s* in the range 0.35–0.37.

The improvement of the solubility of the polyamides is still one of the main key points to overcome. In this sense, the main strategy consists of decreasing the rigidity of the polymer backbone, thus reducing the interchain interactions by the inhibition of the chain packing. The introduction of molecular asymmetry, flexible units, or bulky pendant groups are some of them. In this regard, Sadavarte et al. [28] described the phosphorylation condensation of 4-pentadecylbenzene-1,3-diamine with four commercially available aromatic acids (biphenyl-4,4′dicarboxylic acid, 4,4′-oxybisbenzoic acid, terephthalic acid and isophthalic acid). They obtained medium molecular mass polymers, showing constitutional isomerism, with improved solubility in organic solvents due to the pendant dodecyl chains.

Damaceanu et al. [29] also improved the solubility of polyamides by introducing asymmetry in the polymer structure. Their group produced high molecular mass polyamides, synthesized from asymmetric diamines with phenoxy-substituted benzophene segments, using some aromatic diacid chlorides containing ether, hexafluoroisopropylidene or diphenylsilane groups.

Also, previously, Ueda [30] and Gentile and Suter [31] reviewed the constitutional isomerism in polyamide structures regarding constitutional order, theory, and polyamide structure.

### 3.3. Aromatic Polyamides with Spherical-Like Structures

Aromatic polyamides show outstanding mechanical, thermal, and chemical properties as a result of their linear structure and their interchain hydrogen bonds, and thus they are considered high performance polymers. However, they are also highly insoluble in organic solvents, intractable and cannot be melt-processed. The inhibition of the amide-amide linkages to increase their solubility and make them easier to melt can be achieved by means of introducing asymmetric or bulky pendant groups, or by modification of the polymer architecture. The introduction of dendritic structure, or sphere-like geometry, inhibit the interchain interactions, leading to processable high performance polymers of known molecular mass, spherical, defect free and monodisperse, with low solution viscosity, poor mechanical properties, and good solubility [32]. These properties enable aromatic polyamides to be used in biological and medical applications, and for novel applications related to their encapsulation characteristics, chromatography, catalysis, or self-assembly.

Spherical-like aromatic polyamides can be obtained by different procedures. Dendrimers are prepared either by convergent growth (reaction of preformed dendrons with a central core molecule) or divergent growth (slow addition of building blocks of low molecular mass starting from a polyfunctional molecule, the core) by tedious multiple-steps procedures [33]. The preparation of hyperbranched polymers is much easier, usually following a one-pot procedure under conventional polymerization reactions; and they are neither monodisperse or defect-free. In addition to the dendritic (D) and terminal (T) units present in perfect dendrimers, hyperbranched polymers also have linear (L) units, being their structure monomer-dependent. An overview about the synthesis, properties and applications of these types of aromatic polyamides was published by Scholl et al. [34] and Jikei and Kakimoto [32].

A more recent work by Matsumoto [35] reported the preparation of aromatic polyamide dendrimers, using a divergent approach, up to the sixth generation with a polyhedral oligomeric silsesquioxane (POSS). An eight-functional core was prepared using octa(*n*-propylamine)-POSS and an AB_2_ building block using 3,5-bis(trifluoroacetamido)benzoic acid and deprotected with hydrazine to yield the G1 dendrimer. The rest of the dendrimers (synthesized in a similar way) were obtained in high yields without tedious purification. These organic-inorganic hybrid nanoparticles have potential application in nanoscience as fluorescent nanofillers with controlled functionality, shape and size.

Tsushima and coworkers [36] also reported a much easier procedure to isolate dendrimers compared to traditional methods. They performed a one-pot multistep synthesis by preparing dendritic aromatic polyamide dendrons on a glycine-modified Wang resin as the solid support. Any unreacted chemicals can be removed by washing the resin with solvents, as the propagating dendrons are attached to the insoluble support.

Aromatic and semiaromatic amine-terminated hyperbranched polyamides and carboxylic acid-terminated hyperbranched polyamide-esters were synthesized by Shabbir and coworkers [37]. The former were prepared by low temperature polycondensation of an aromatic triamine (1,3,5-tris(4′-aminophenylcarbamoyl)benzene) (B_3_ monomer) with different A_2_ diacid chlorides (terephthaloyl chloride, isophthaloyl chloride, sebacoyl chloride and adipoyl chloride) to render hyperbranched polyamides soluble in polar aprotic solvents, with glass transition temperatures (*T*_g_) between 138 and 198 °C and molecular masses in the range of 1.3 × 10^4^–2.7 × 10^4^.

**Scheme 6:**
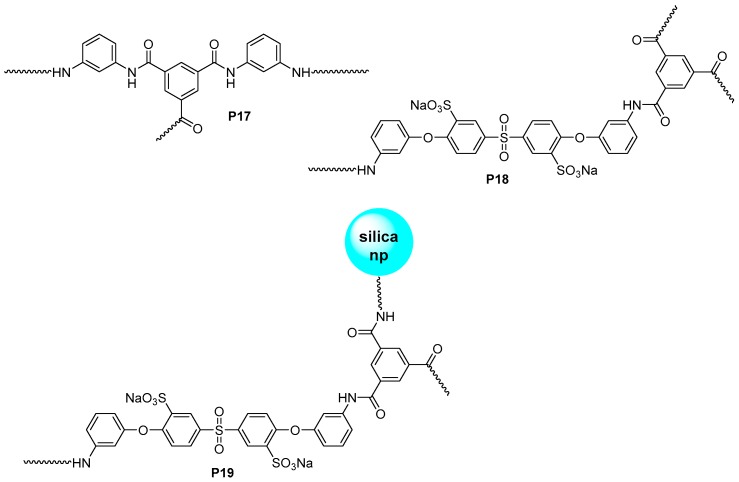
Hyperbranched aramids.

Fluorescent porous hyperbranched aramids with different terminal functional groups through an A_2_ + B_3_ approach have been synthesized by Hu [38]. He used 1,3,5-tri(4-carboxyl phenyl) benzene and *p*-phenylenediamine to prepare polyamides with strong blue fluorescence with potential applications in storage, separation, catalysis and light emitting. Examples of other applications of hyperbranched polyamides are their use in reverse osmosis membranes [39], (Scheme 6, P17–P19), in applications related to their magnetic properties due to the complexation of hyperbranched polyamides with a metal [40], to their electrochromic properties after the introduction of electroactive units [41], or the improvement of thermal properties of composites grafted with hyperbranched polyamides [42,43].

## 4. Aromatic Polyamides with Selected Properties and Applications

### 4.1. Polyamides with High Thermal Stability and Flame-Retardant Properties

Aramids are considered a high performance polymer mainly for two reasons, for their outstanding mechanical performance and for their thermal stability and flame resistance. In this section, we describe some of the novel structures reported in the literature during the last years. Polymers with flame retardant properties usually contain halogens in their structure and some of them will also be described later.

**Scheme 7:**
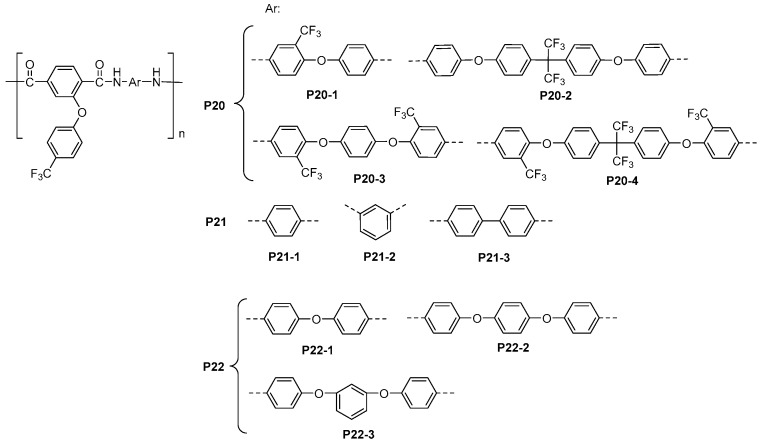
Fluorinated aramids.

In a first group, several authors have studied the synthesis of good thermal stability fluorinated polyamides. Jiang et al. [44] reported on the preparation of fluorine containing polyamides obtained from polycondensation of 2-(4-trifluoromethylphenoxy)terephthalic acid with four trifluoromethyl-substituted aromatic bis(ether amine)s (Scheme 7, P20). Polymers had *T*_g_s between 189 and 214 °C, and 10% mass loss temperatures ranging from 475 to 483 °C. Ghaemy et al. [45] describe the preparation of a series of fluorescent anthraquinone-quinoxaline containing polyamides, prepared from an aromatic diamine, 2,3-bis(4-(4-amino-2-(trifluoromethyl) phenoxy)phenyl)naphtho[2,3-f]quinoxaline-7,12-dione. Polyamides had η_inh_ between 0.39 and 0.62 dL/g, *T*_g_s between 230 and 323 °C, and 10% mass loss temperatures in the range of 362 to 433 °C in N_2_. Shockravi et al. [46] present a series of organic-soluble poly(ether-amide)s bearing sulfoxide and electron withdrawing trifluoromethyl group, starting from bis(ether-amide) monomer, 2,2′-sulfoxide-bis[4-methyl(2-trifluoromethyl)-4-aminophenoxy) phenyl ether], and several aromatic acids. The polymers could be cast into flexible and tough films from DMA solutions, with *T*_g_s between 160 and 220 °C. In addition, 10% mass loss temperatures were recorded in the range of 400 to 505 °C in N_2_ and from 380 to 480 °C in air atmosphere. Also, Damaceanu et al. [47] prepared two different aromatic fluorinated polyamides. One of the polyamides contained in the main chain 1,3,4-oxadiazole and naphthalene moieties. The other derived from an asymmetrycal diamine containing a phenoxy-substituted benzophenone. The obtained polyamide films showed excellent thermal stability, with starting decomposition temperatures above 400 °C. Finally, Jiang et al. [48] synthetized 9,9-bis[4-(chloroformylphenoxy)phenyl]xanthene, from which the authors obtained aromatic polyamides with ether and bulky xanthenes groups, by means of low temperature polycondensation with several aromatic diamines. Concerning thermal properties, the *T*_g_s varied between 236 to 298 °C, with decomposition temperatures at 10% mass loss between 490 and 535 °C in N_2_, and up to 515 °C in O_2_.

**Scheme 8:**
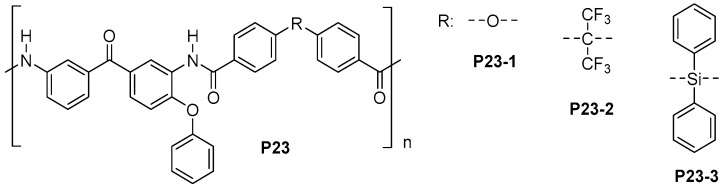
Aramids with substituted benzophenone in the main chain.

Another investigation line is focused in the use of substituted benzophenone in the main chain of aramids. As noted previously, Damaceanu et al. [29] prepared and characterized several aromatic polyamides containing pendant phenoxy motifs in benzophenone sub-structure in the structural unit, with thermal stability up to 385 °C and *T*_g_s ranging from 225 to 256 °C (Scheme 8, P23).

Patil et al. [49] prepared polymers reacting the monomer having aromatic–aliphatic amide and ether linkages, bis-[(4-aminobenzyl)-4-benzamide] ether, with different mole proportions of IPC or TPC. Differential scanning calorimetry (DSC) analysis of these polyamides showed *T*_g_s in the range of 197 to 204 °C, and they showed no mass loss below 336 °C when analyzed by thermogravimetry.

Shabbir et al. [50] describe the polycondensation of 4-hydroxy-2,6-diaminopyrimidine with different diacid chlorides, to obtain semiaromatic hyperbranched and carboxylic acid terminated aromatic polyamide-esters. The obtained amorphous polymers had η_inh_ ranging between 0.21 dL/g and 0.28 dL/g and had excellent thermal stability with 10% mass loss at temperatures from 346 to 508 °C. As seen previously, Sadavarte et al. [28] also analyzes the polycondensation of a diamine (pentadecylbenzene-1,3-diamine) with different commercial aromatic diacids to obtain aramids with pendant pentadecyl chains. Thermal properties reported include 10% mass loss values up to 460 °C (in N_2_), high for aramids containing aliphatic chains, and *T*_g_s values varying between 169 to 215 °C.

Mallakpour et al. [51] employed a chiral diacid, (2*S*)-5-[4-(4-methyl-2-phthalimidylpentanoyl-amino)benzoylamino]isophthalic acid, in a polycondensation process with aliphatic and aromatic diisocyanates. The polyamides obtained presented interesting thermal properties (5% mass loss temperature up to 355 °C in N_2_).

Flame-retardancy polyamides were also analyzed by several authors. Also, the group of Mallakpour et al. [52] describe the synthesis of aromatic polyamides with flame retardancy properties, using conventional heating as well as microwave irradiation. The polymers were prepared using a diacid monomer with a l-phenylalaninetetrabromophthalimide group.

**Scheme 9:**
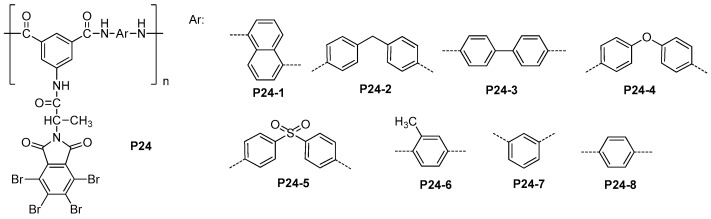
Aromatic polyamides with pendant tetrabromophthalimide in the side chain.

To improve thermal resistance and flame retardancy properties, halogens as bromine and chlorine are usually employed in the formulation of all kind of polymers, also in aromatic polyamides. For example, Rafiee et al. [53] describe the preparation of flame-retardancy polyamides, with tetrabromophthalimide in the side chain (Scheme 9, P24). Polyamides produced presented good thermal stability (10% mass loss up to 475 °C in N_2_), inherent viscosities (η_inh_) between 0.36 and 0.63 dL/g, and were also biodegradables.

In addition to the heat and chemical resistance and enhanced solubility imparted by halogenated groups in polyamides, organofluorine compounds also possess a unique combination of characteristic features such as water and oil repellency, nonstick and electrical insulation properties, indispensable for specific applications in electronics, coating fields and so on. Nano- and submicron sized polymer particles have prepared for certain applications in these fields. In this regard, Yoshioka [54] presented a precipitation polymerization method, from 4,4′-diphenyldicarbonyl chloride and 2,2-bis(4-aminophenyl)hexafluoropropane, to prepare nano-sized aromatic polyamide particles containing trifluoromethyl groups, using a pyridine containing dioxane. As the pyridine content increased, the particle diameter decreased from 500 to 150 nm, and so did the molecular mass, the yield, and the thermal properties. The degrees of crystallinity were low and the thermal decomposition temperatures were above 457 °C. In a later research, the same author and Tashiro [55] described the preparation of polyamide nanofibers, from 4,4′-diphenyldicarbonyl chloride and 2,2′-bis(trifluoromethyl) benzidine in dioxane solution with a water content of 9% vol. The structure of the fibers consisted of intimately intertwined nanofibers with an average diameter of 55 nm. The strong interactions between the polymer and the solvent in the reaction solution resulted in the formation of a gel. During the fiber formation process, the product in the reaction solution transitioned from particles into fibers with an increase in molecular mass. The degree of crystallinity and the hydrogen and the interchain bonding of the polymers increased with the reaction time, while the morphology became less regular.

### 4.2. Mechanical Properties

As a class of high-performance materials, wholly aromatic polyamides exhibit superior properties, such as mechanical resistance. In this section, we describe research works focused in the mechanical behavior, i.e., in the determination of parameters such as elongation at break and Young’s modulus.

**Scheme 10:**
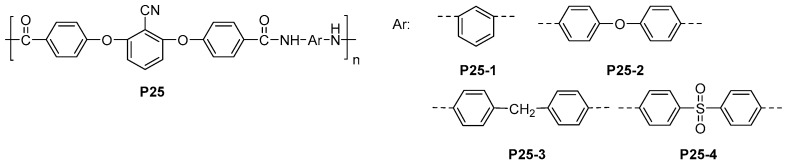
Aramids with cyano groups.

Tan et al. [56] describe the synthesis of polyamides containing phthalazinone from the acid monomer 4-[4-(4-carboxyphenoxy)-naphthyl]-2-(4-carboxyphenyl)phthalazin-1-one, with η_inh_ ranging from 0.54 to 0.69 dL/g. Films derived from these materials had good mechanical properties (elongational break up to 11.4% and tensile strength values from 63.9 to 81.6 MPa). Previously we presented the work of Jiang et al. [44] describing the polycondensation of 2-(4-trifluoromethylphenoxy)terephthalic acid with trifluoromethyl-substituted aromatic bis(ether amine)s to obtain amorphous fluorine-containing polyamides. The mechanical properties of the cast films, prepared from DMA, had tensile moduli from 2.7 to 3.2 GPa, elongations at break between 6% and 9% and tensile strengths up to 115 MPa. Yu et al. [57] described aromatic diamines with cyano groups from the monomer 2,6-bis(4-chloroformylphenoxy)benzonitrile. Cast films from *N*,*N*-dimethylformamide (DMF) showed tensile strength values from 79 to 93 MPa, Young’s moduli from 1.7 to 2.6 GPa, and elongation at break from 9 to 15%, indicating they are strong in mechanical properties (Scheme 10, P25).

Several soluble and amorphous aramids were obtained from semifluorinated aromatic diamines by Bera et al. [58]. The polyamides had η_inh_ from 0.42 to 0.63 dL/g and cast films showed good mechanical properties (elongations at break up to 25%, modulus of elasticity up to 1.81 GPa and tensile strengths values up to 88 MPa). Diamines employed included 4-bis[3′-trifluoromethyl-4′ (4″-amino benzoxy)benzyl]biphenyl; 4,4″-bis(aminophenoxy)-3′3″-trifluoromethyl terphenyl; 1,3-bis[3′-trifluoromethyl-4′ (4″-amino benzoxy)benzyl]benzene; 2,6-bis(3′-trifluoromethyl-*p*-aminobiphenyl ether)pyridine; and 2,5-bis(3′-trifluoromethyl-*p*-aminobiphenyl ether)thiophene with 5-*t*-butyl-isophthalic acid.

Jiang et al. [48] prepared a series of aramids from 9,9-bis(4-hydroxyphenyl)xanthenes and aromatic diamines. The polymers, with ether and bulky xanthenes groups (η_inh_ from 0.72 to 0.98 dL/g), showed good mechanical properties (tensile strengths ranging from 82 to 106 MPa, elongations at break from 10 to 25%, and initial moduli from 2.0 to 2.8 GPa). Sheng et al. [59] also described the preparation of aramids with xanthene moietis, starting from 9,9-bis[4-(4-carboxyphenoxy) phenyl]xanthene and 9,9-bis[4-(4-aminophenoxy) phenyl]xanthene. The polymers had η_inh_ from 0.82 to 1.32 dL/g and gave flexible and transparent films by casting (moduli from 2.15 to 2.63 GPa, tensile strengths ranging from 86 to 109 MPa and elongations at break from 13 to 22%).

Zou et al. [60] prepared cast films from aromatic polyamides containing 4-aryl-2,6-diphenylpyridine moieties and pendant fluorinated phenoxy groups, showing good mechanical properties, such as tensile moduli between 2.35 to 2.87 GPa, elongations at break between 5.3 to 9.5% and tensile strengths between 72.5 to 87.3 MPa. Li et al. [61] prepared poly-*p*-phenylene-benzimidazole-terephthalamide fibers by wet spinning, using a two-step drawing-annealing process to achieve high orientation degree, improving the strength of the hydrogen bonding. Using this procedure, tensile strength increases up to 15% respect to fibers produced using one-step drawn. Finally, Abronin et al. [62] showed a theoretical modelling of the analysis of the influence of hydrogen bonds in aromatic polyamides on technological processing methods to produce heavy-duty aramid yarns. Quantum-chemical calculations of model fragments in conjunction with vibrational spectra in the infrared range have shown that, depending on the hydrogen bonds between the benzimidazole groups, “self-orientation” processes emerged caused by the increase in energy of the hydrogen bonds in the aramid yarns and fibers. This explained the uniquely high values describing mechanical properties of the yarns and their performance characteristics in cloths, plastics, and spool products.

The mechanical parameters described in the reviewed papers are summarized in Table 2.

### 4.3. Sensing and Extraction Applications

The development of selective and sensitive materials for sensing anion, cation and neutral molecules is a topical research area which has gain a lot of interest in the last years. For this reason, the use of polyamides and their extraction applications is currently under investigation. In this review, we collect the recent works concerning this research topic.

For example, San-José et al. [63] present an aromatic diamine for sensing applications, with a colorimetric diacid monomer with the urea binding site. The monomer changes color from colorless to blue, brown or yellow depending on the addition of *meta*, *para* or *ortho*-phenylenediamine, respectively, remaining inactive against other amines or diamines.

**Scheme 11:**
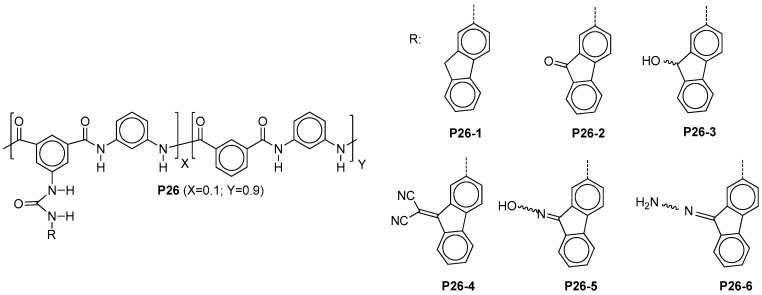
Aramids with pendant fluorene-derivative motifs.

Colorimetric acid responsive coated fibers and films were analyzed by Trigo-López et al. [64] Materials were prepared by condensation or addition monomers having the azobenzene group (dye moiety) and *N*,*N*-dimethylamino motifs. Cotton commodity fabrics and high-tech aromatic polyamides were modified to analyze the applicability of these materials. Colorimetric sensors, with *pK_a_*s in water from 1.78 to 0.5 and in air from −1.5 to −3.9 were developed, with color change from yellow/blank to red/purple in presence of acid media. Aromatic polyamides with pendant fluorescent chemical structures have been also analyzed by Estévez et al. [65]. In this work, copolyamides with lateral fluorene-derivative moieties anchored to the main chain through a urea group were prepared, chemically modifying the fluorene moieties to obtain different fluorescent behaviors. Then, materials could be prepared tunable fluorescence properties (Scheme 11, P26). Following this line, Barrio-Manso et al. [66] prepared luminescent polyamides, starting from a fluorescent dipicolinic acid derivative. The materials behaved as effective fluorescence sensors for relevant cations, such as Cr(VI), Fe(III) and Cu(III). In the work of Gómez-Valdemoro et al. [67], aromatic polyamides and copolyamides with pendant triazole heterocycles were described. The aramids exhibited high *T*_g_s (up to 375 °C) and behaved as sensory materials for heavy metal cations. Polyamides showed capability of cation extraction from aqueous media (close to 100% in Hg(II)). Finally, Yen et al. [68] presented triarylamine-based aramids with secondary amine moieties, prepared and utilized as electrochemical detectors for pyridines.

### 4.4. Film, Membranes and Transport Properties

Crosslinked aromatic aramids are usually the active layer of commercial reverse osmosis membranes for water treatment and desalination processes. These membranes are usually prepared depositing a non-porous active monolayer of aromatic polyamide, by interfacial polymerization, on a micro or ultrafiltration polysulfone membrane directly prepared over a woven/non-woven polyester fabric, which acts as a mechanically strong support. Likewise, the separation of gases is a promising clean technology for obtaining pure gases from their mixtures, for example nitrogen and oxygen from air, and also for cleaning of petrochemical-derived products, e.g., synthetic gas, and decontamination of industrial gases. Gas separation is carried out with polymeric materials using non-porous dense membranes, i.e., films, prepared by conventional techniques, e.g., casting from solution or spin coating. In this section, we collect the principal research works related to this interesting topic, which has been extensively studied the last years.

**Scheme 12:**
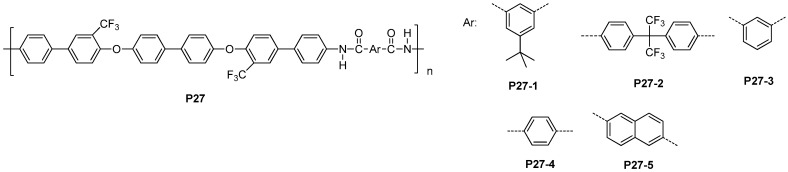
Aromatic polyamides for gas separation.

**Scheme 13:**
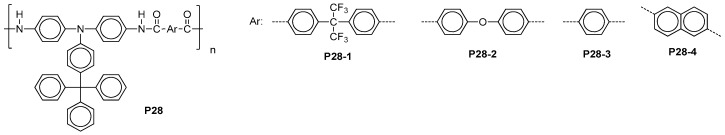
Polyamides with bulky pendant group for gas separation.

**Scheme 14:**
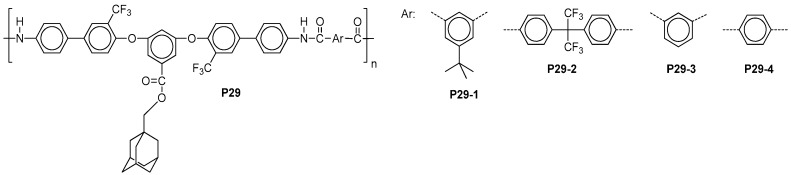
Aramids with pendant bulky adamantyl-moieties.

**Scheme 15:**
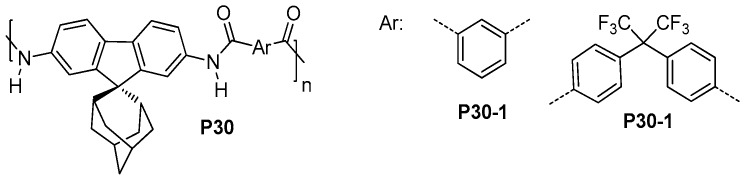
Aramids with rigid spiro-moieties in the main chain.

In relation with gas separation membranes, Bandyopadhay et al. [69], obtained poly(ether amide)s based on 4,4-bis-[2′-trifluromethyl 4′-(4″-aminophenyl) phenoxy]-biphenyl monomer. The obtained membranes showed a good permselectivity for O_2_ and N_2_ gas pair (P_O_2__/P_N_2__ = 7.1) and CO_2_ over CH_4_ (P_O_2__/P_CH_4__ = 30.3). (Scheme 12, P27). Also, Bisoi et al. [70] analyzed the gas permeation properties of several gases through membranes based on polyamides produced by the polycondensation reaction of 4,4′-diamino-4-trityl aniline with commercial diacids (Scheme 13, P28), observing that incorporating a trityl group into the polymer backbone resulted in P_CO_2__ and P_O_2__ values as high as 141 and 33 Barrer. Smith et al. [71] studied the transport properties of membranes to separate H_2_, N_2_, O_2_, CH_4_ prepared by thermally rearranged polymers. These polymers have reactive groups. These groups undergo rearrangements upon thermal treatment of the membranes, usually increasing the free volume of the materials, which is a key parameter in gas membrane performance. Smirnova et al. [72] studied the gas separation properties of membranes prepared from sulfonate containing aromatic polyamide and aliphatic polyamines, reporting on the relationship between the nature of polyamine, the composition of the interpolymer complex, and the degree of transformation in it in the gas separation characteristics of the membranes. Bera et al. [73,74] reported the synthesis of a diamine monomer, 1-adamantylmethyl[3,5-bis-{2′-trifluoromethyl-4′-(4″-aminophenyl)phenoxy}]benzoate and its polymerization with four different diacids (Scheme 14, P29). The permeability of CH_4_, N_2_, O_2_ and CO_2_ through these membranes was investigated at three different temperatures (35, 45 and 55 °C) under an applied upstream pressure of 3.5 bar. The gas transport properties were correlated with the polymer structures with respect to their *T*_g_, chain packing and dielectric constant values. Similar studies were with aromatic polyamides containing 1-adamantanemethoxy substituted triphenylamine and the obtained membranes to analyze gas separation processes. Plaza-Lozano et al. [75] reported the synthesis of polyamides and polyimides with high *T*_g_ values above 350 °C and onset degradation temperatures higher than 450 °C. Polymers were prepared from a rigid diamine monomer, namely spiro-(adamantane-2,9′(2′,7′-diamino)-fluorene) (Scheme 15, P30). The values of permeability and selectivity showed well-balanced gas productivity, in particular for the CO_2_/CH_4_ separation. Flourinated polyamides, analyzed by Damaceanu et al. [47], showed good permeability properties against different gases, as a function of the structural variations in the polyamide repeating unit.

The correlation between gas and vapor separation characteristics of aromatic polyamides with the bulkiness of the aramids was studied theoretically. Chang et al. [76], adopted several simulation techniques, such as molecular dynamics or the Monte Carlo method, to analyze free volume, fractional accessible volume and the size and shape distributions of the free volume, showing that bulky groups in the membranes promote the formation of a continuous and larger free volume. Finally, a correlation between theoretical and experimental data was observed.

Regarding reverse osmosis membranes, as exposed previoulsy, Park et al. [39] prepared hyperbranched aromatic polyamide-grafted silica and disulfonated 4,4-bis(3-aminophenoxy)phenyl sulfone with the purpose of enhancing the chlorination resistance in desalination processes. Lee et al. [77] also analyzed thin film composite membranes for salt rejection. The layer was obtained via interfacial polymerization of two monomers 1,3-phenylene diamine and trimesoyl chloride, with a thickness about 200 nm. The membrane thickness as a function of polymerization time and monomer concentration was investigated. The authors measured the zeta potential of the membrane surface that usually faces the membrane support, which was negatively charged for all pH values above 4.0.

The modification of commercial reverse osmosis membranes has also been extensively studied. Xu et al. [78] prepared polyethylenimine with different molecular masses to alter the surface charge of an aromatic polyamide reverse osmosis membrane, that was reversed (isoelectric point changed from pH 3.1 to pH 8.0). The surface hydrophilicity was also improved, showing high anti-fouling properties to the positively charged pollutants. Wei et al. [79] and Zhang et al. [80] modified commercial membranes adding 3-allyl-5,5-dimethylhydantoin by a free-radical graft polymerization process, finding, for example, that the chlorinated grafted membranes had remarkable sterilization effects on Escherchia Coli and good antimicrobial fouling. For example, Hu et al. [81] and Liu et al. [82] modified commercial aromatic polyamides reverse osmosis membranes by a surface treatment using a glutaraldehyde aqueous solution and in a second step, a polyvinyl alcohol solution. The authors showed that a covalent bonding of polyvinyl alcohol macromolecules on the pristine membrane occurs, increasing surface roughness, hydrophicility and also improving the membrane antifouling property against bovine serum albumin, sodium dodecylsulfate and dodecyltrimethyl ammonium bromide. Wu et al. [83] modified a commercial aromatic polyamide thin-film reverse osmosis membrane with a thermo-responsive copolymer, poly(*N*-isopropylacrylamide-*co*-acrylamide). The modified membranes changed their hydrophicility as a function of temperature, then increasing the resistance to permeation. To conclude, commercial nanofiltration membranes have been also modified using, in this case, shape-persistent dendritic molecules [84]. In this case, amphiphilic dendrimers modified the membrane directly by percolation. The results showed an accumulation of dendrimers inside the membrane, with a pure aramid coating on top.

The porous structure of reverse osmosis membranes was studied by Yan et al. [85]. Homemade reverse osmosis membranes were analyzed by scanning electron microscopy and transmission electron microscopy, showing protuberances and ridges with interconnecting cavities and tunnels, thus demonstrating the Carother’s theory about the formation of pores during the interfacial polymerization process.

We end this section with the preparation of proton exchange fuel cell membranes. Wang et al. [86] describe the synthesis of sulfonated aromatic polyamides with nitrile groups. The aramids were prepared from 6-bis(4-aminophenoxy)benzonitrile, 5-sulfoisophthalic acid sodium salt and terephthalic acid. The materials presented ion exchange capacity about 1.87 mEquiv/g and proton conductivity of 141 mS/cm at 80 °C.

### 4.5. Optically Active and Fluorescent Polyamides

As it has been explained in the introduction, one of the main research key points is related to the analysis of the properties of polyamides incorporating new chemical functionalities in their structure. Among these properties, optical properties, such as fluorescence and/or the use of polyamides as light-emitting diodes or organic light-emitting diodes devices are specially interesting nowadays. In this section, we review the research lines carried out in this investigation area during the last years.

**Scheme 16:**
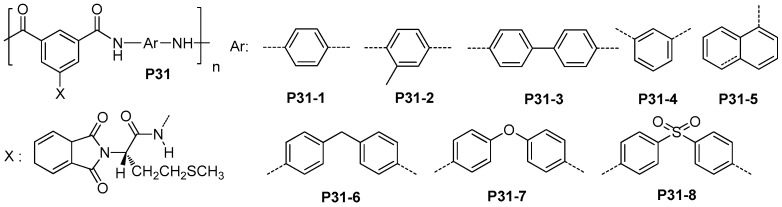
Aramids with pendant optically active groups.

Mallakpour et al. [51,52,87,88,89,90,91] developed optically active monomers based on optically active aminoacid cores, such as l-leucine (see example in Scheme 16, P31). Interestingly, extensive use of green chemistry (ionic liquids as solvents) and non-conventional heating techniques (microwave radiation) were reported. The polycondensation proceeded with shorter times and higher yields than with conventional heating. The polymerizations were carried out following the conventional condensation of diacid or diacid chlorides with diamines and were also accomplished with condensation of diacids with diisocyanates. In addition to the good thermal properties, the polyamides synthetized by Rafiee and Mallakpour [53] mentioned previously (Scheme 9, P24) were also optically active due to the chiral center of amino acid moiety, and the bulky side chain disturbed the interchain interaction, reducing packing efficiency and crystallinity, and making them readily soluble in solvents such as NMP, DMA, dimethyl sulfoxide (DMSO) and DMF. All these features make these polymers potentially useful as chiral stationary phase in chromatography technique and for separation of racemic mixtures.

**Scheme 17:**
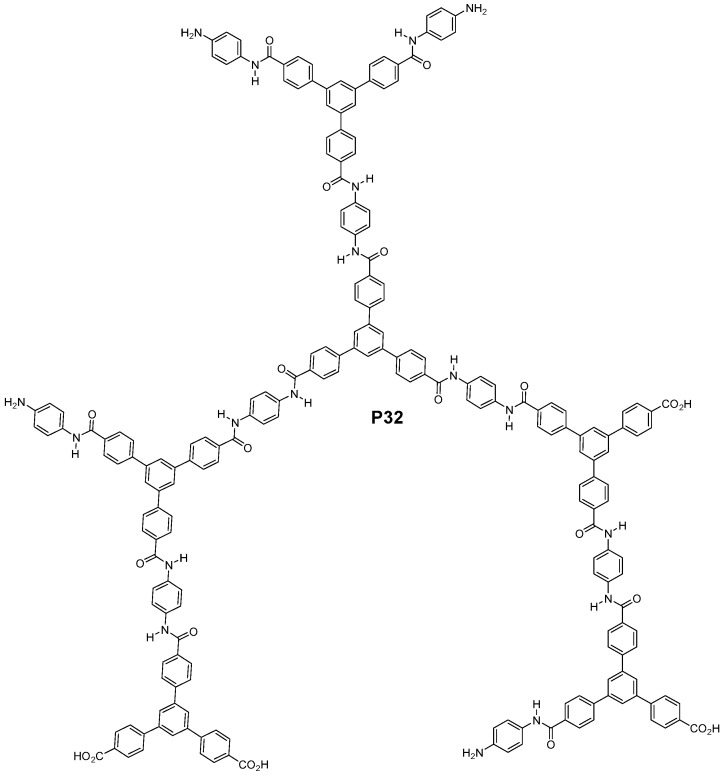
Fluorescent hyperbranched aramids.

**Scheme 18:**
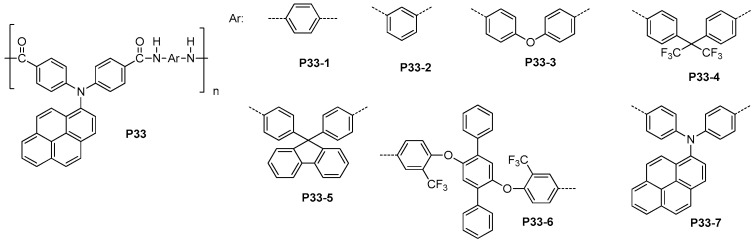
Fluorescent and electrochromic aramids with pyrenyl motifs.

Using another approximation, as showed previously, Hu et al. [38] obtained two porous hyperbranched poly(1,3,5-tris(4-carboxyphenyl) benzene *p*-phenylenediamine) amides with different terminal functional groups using 1,3,5-tris(4-carboxylphenyl) benzene and *p*-phenylenediamine as raw material, by means of regulating the mole ratio of the monomers (Scheme 17, P32). Both polymers were soluble in DMSO and DMF. Their DMSO solutions exhibit strong blue fluorescence, especially for the amino terminated polymer. While in DMF solution, the two polymers emit strong green fluorescence, and showed potential to be employed in the areas of storage, separation, catalysis, and light emitting. Kung et al. [92] prepared aramids with pyrenylamine motifs in the main chain from the diacid monomer *N*,*N*-di(4-carboxyphenyl)-1-aminopyrene. The polyamides were soluble and could be cast into tough films. NMP solutions exhibited fluorescence maxima around 455–540 nm with remarkable quantum yields (up to 56.9%), and showed solvatochromism. Their films showed reversible electrochemical redox behavior exhibiting remarkable electrochromic behavior (colorless, neutral state; purple, oxidized state; yellow, reduced state) (Scheme 18, P33).

**Scheme 19:**
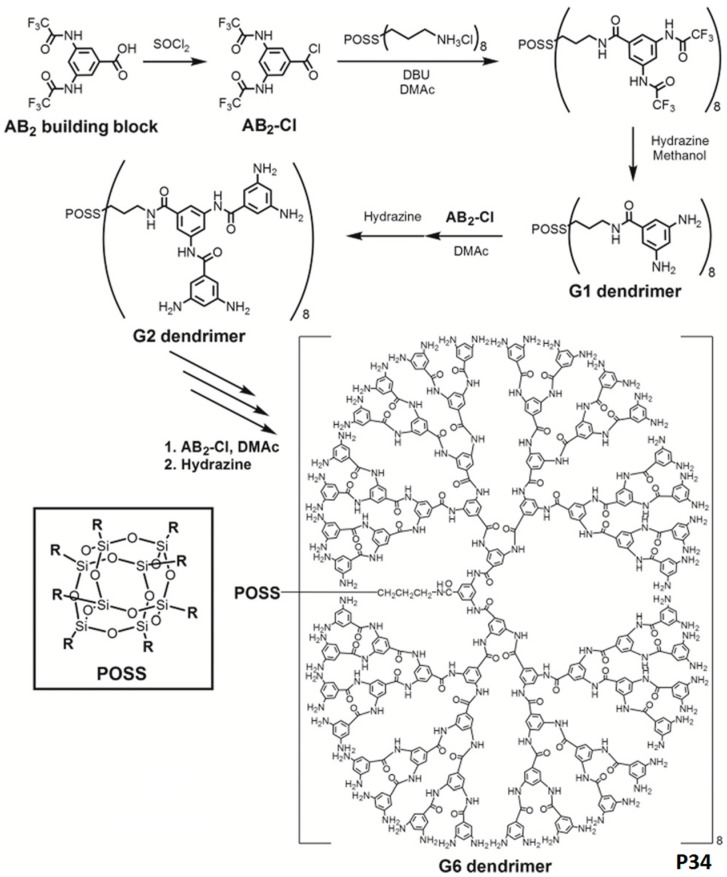
Fluorescent aromatic dendrimers (Reproduced from ref. [35] with permission from The Royal Society of Chemistry).

Lee et al. [93] studied a series of *para*-linked aromatic polyamides having η_inh_ of 0.51 to 0.91 dL/g, that were prepared from 2-trifluoromethyl-4,4′-diaminodiphenyl ether, 2-cyano-4,4′-diaminodiphenyl ether, 2-trifluoromethyl-4,4′-diaminodiphenyl sulfide, and 2,2′-bis(trifluoromethyl)-4,4′-diaminodiphenyl sulfide. All of the synthesized polyamides presented low refractive indices (*n*) in the range of 1.616 to 1.661 and low birefringence (Δ) in the range of 0.010 to 0.031 with slightly higher *n*_xy_ than *n*_z_, indicating that the polyamides films were slightly anisotropic. As explained previously, Matsumoto et al. [35] synthesized aromatic polyamide dendrimers possessing POSS cores. Octa(*n*-propylamine)-POSS and 3,5-bis(trifluoroacetamido)benzoic acid were prepared as an eight-functional core and an AB_2_ building block, respectively. Condensation of the core with the AB_2_ building block, followed by deprotection via transamidation with hydrazine, was conducted to provide the generation 1 dendrimer. The other dendrimers were prepared in a similar manner. The trifluoroacetamide-terminated dendrimers showed specific fluorescence emission at 445 nm under 330 nm irradiation and the emission intensity increased with increasing the generation number (Scheme 19, P34). Li et al. [94] prepared aromatic polyamides from a diamine with a trifluoromethyl pendant group, 1,4-bis((4-amino-2-(trifluoromethyl)phenoxy)methyl)cyclohexane, and different dicarboxylic acids, namely isophthalic acid, 2,2-bis(4-carboxy-phenyl)hexafluoropropane and 4,4′-oxydibenzoic acid. The polymers had η_inh_ from 1.85 to 2.36 dL/g, and their films were transparent with ultraviolet cut-off wavelength in ranging 326–333 nm, making them suitable for advanced microelectronic applications. To conclude this section, Nechifor et al. [95] prepared aromatic polyamides with photosensitive coumarin pendant groups from the diacid monomer 6,6′-methylenebis{2-oxo-8-{2-[(2-oxo-2H-chromen-7-yl)oxy]acetoxy}-2H-chromene-3-carboxylic acid} and a number of aromatic diamines. The polymers were readily soluble in organic solvents, and had η_inh_ from 0.40 to 0.87 dL/g. Ultraviolet irradiation of the polymer films gave rise to crosslinked materials through a [2*π* + 2*π*] photocycloaddition at the carbon-carbon double bond of coumarin motifs.

### 4.6. Electrochemical and Electrochromic Properties

Electrochromic materials are technological materials for the preparation of tunable windows, variable reflectance mirrors, and electrochromic displays. These materials can be oxidized or reduced reversibly, partially or completely, with a concomitant color change. Accordingly, the color change can be easily tuned. The use of high performance polymers that can be easily processed by casting or spin-coating, and at the same time withstand harsh environments or thermally and mechanically demanding conditions, is highly appealing. For these reasons, the synthesis and characterization of aromatic polyamides with electrochemical and electrochromic properties has gained considerable interest in latest years.

**Scheme 20:**
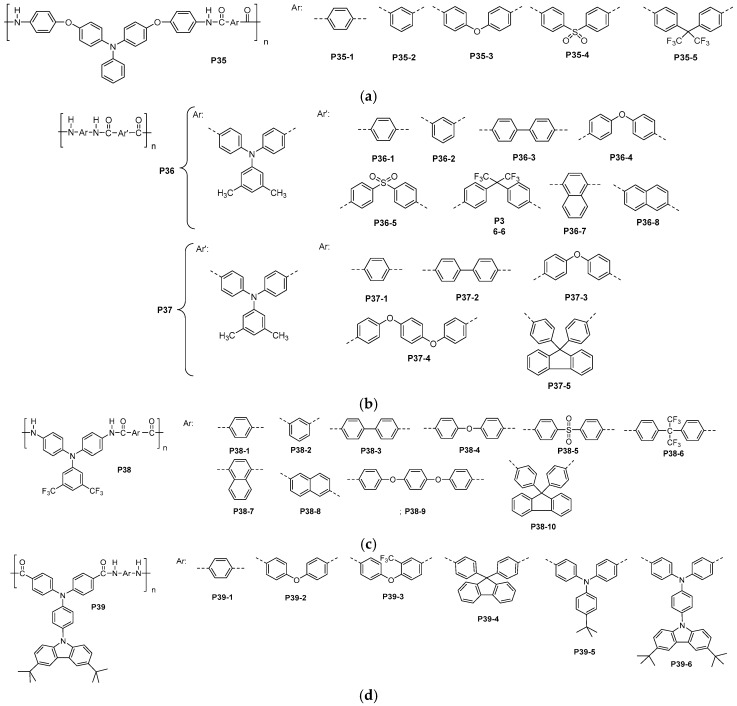

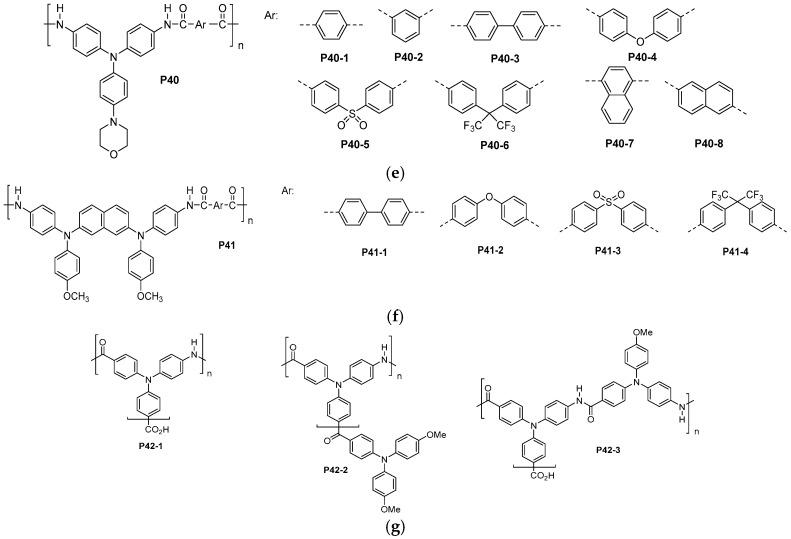
Electroactive aramids with the following electroactive moieties: (**a**) triphenylamine; (**b**) 3,5-dimethyltriphenylamine; (**c**) 3,5-bistrifluoromethyltriphenylamine; (**d**) triphenylamine with pendant carbazole; (**e**) morpholinyl-substituted triphenylamine; (**f**) 2,7-bis(diphenylamino)naphthalene; (**g**) triphenylamine(hyperbranched aramids).

In this section of our review we summarize the works published in this research field by Hsiao and coworkers [96,97,98,99,100,101,102,103,104,105,106,107,108,109,110,111,112,113,114,115,116,117,118,119,120,121,122,123,124,125], Sun et al. [126] and He et al. [127]. Electrochromic aramids are principally based on the polymerization of monomers that contain sub-structures that are the responsible for the reversible electrochemical oxidation processes accompanied with strong color changes of the polymer films prepared by casting, spin-coating or electropolymerization. These electroactive groups are mainly triphenylamine (TPA), *N*,*N*,*N*′,*N*′-tetraphenyl-1,4-phenylenediamine (TPPA), diphenylpyrenylamine, bis(diphenylamino)naphthalene, diphenylfluorenylamine, and carbazole (Table 3). Scheme 20 shows examples of the polyamide structures. The electrochemical behavior imparted by these groups was tuned by electron pumping or withdrawing groups (Table 3) to render color change from colorless to all the chromatic spectra (visual to near infrared), usually with high coloration efficiency, contrast ratio, optical transmittance change, and rapid switching time, applying affordable potentials. For instance, TPA-based aramids with a piperidinyl auxiliary group, that are soluble in aprotic polar solvents, gave strong films by casting. These films exhibited well-defined and reversible oxidation waves with onset potentials ranging from 0.27 to 0.35 V (vs. Ag/AgCl; acetonitrile solution). The redox behavior of the colorless films was accompanied by a strong color variation in the range of 0.75 to 1.20 V, from colorless (neutral) to green and deep blue (oxidized forms). The optical transmittance change between the neutral and the fully oxidized states was up to 83% (at 636 nm) [106].

### 4.7. Electrical and Magnetic Properties

**Scheme 21:**
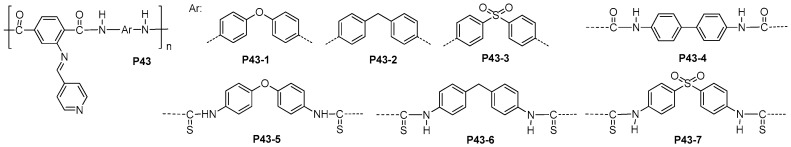
Aramids with having both pyridine and azomethine units.

Although electrical and magnetic properties of polyamides have not been extensively analyzed compared to thermal or mechanical characteristics, a few works related to the study of electrical conductivity or ferromagnetism (useful in memory applications, such as static random access memories) in organometallic complexes or doped aramids have been recently published. Ravikumar et al. [128] studied the electrical conductivity of polyamides prepared from aromatic dicarboxylic acid monomer, 4-pyridylformylimino-*N*-(phenyl,2′,5′-dicarboxylic acid), having both pyridine and azomethine units. The conductivity of the polyamides, when blended with 20% mass of doped polyanilines, was in the range between 3.1 and 4.2 × 10^−3^ S/cm (Scheme 21, P43).

**Scheme 22:**
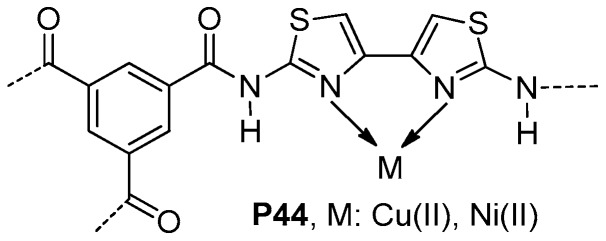
Hybrid ferromagnetic aromatic polyamides.

Magnetic properties, and specially their application as memory devices has been analyzed by several authors. Chen et al. [129] developed sulfonyl-containing aromatic polymers: polyether, polyester, polyamide, and polyimide consisting of a triphenylamine synthetized moiety. Then, the memory behavior of the polyamides was investigated, by choosing the suitable linkage between the electron donor and acceptor, finding tunable memory properties (from insulator to static random access memories characteristics). Following the same investigation line, Huang et al. [130] studied the linkage effect and donor–acceptor effect on memory behavior (from dynamic random access memory, to write once read many memory) of 4-(*N*-carbazolyl)triphenylamine-based polymers, analyzing their memory behaviors. Finally, as shown previously, Ding et al. [40] developed a hyperbranched aromatic polyamide based on the polycondensation of two monomers, one bifunctional and the other trifunctional, 2,2′-diamino-4,4′-bithiazole and trimesoyl chloride, respectively, from which two kinds of hybrid organic-inorganic aramids containing Cu^2+^ and Ni^2+^ complexes were prepared. The hybrid aramids were ferromagnetic polymers (Curie-Weiss temperature of 102 and 53 K, respectively) (Scheme 22, P44).

### 4.8. Inherent Coloration

Most of the applications of the aramids require colored fibers, specifically in those related with apparels, carpets, drapes, etc. The dense molecular packaging, the cohesive energy, the molecular interactions and the chain orientation and crystallinity make these polyamides high thermal and mechanical resistant, but also impair their dyeing properties. Ideal color fastness of fibers is achieved by intrinsically, inherently or self-colored polymers [131]. For this reason, research has been directed into the synthesis of inherently colored polyamides by means of introducing chromophores in their structure. This way, migration of the dye is avoided and an even distribution of the chromophore is achieved due to random copolymerization.

A few studies related to these materials have been carried out. Mohamed et al. [132], synthetized twelve intrinsically orange colored aromatic azopolyamide-hydrazides. Polymers and copolymers were prepared from the reaction of 4-amino-3-hydroxybenzhydrazide and 3-amino-4-hydroxybenzhydrazide with 4,4-azodibenzoyl chloride and 3,3′-azodibenzoyl chloride.

**Scheme 23:**
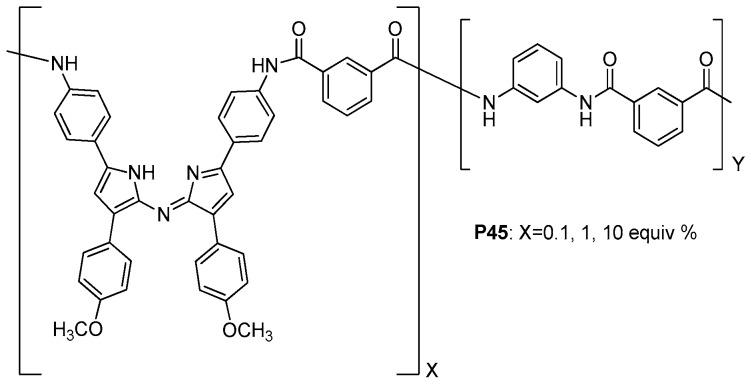
Blue inherently colored aromatic copolyamides.

Blue is the most demanded color in the field of protective clothes. In this regard, Trigo-López et al. [133] prepared three high-performance wholly aromatic polyamides, (Scheme 23, P45), by copolymerization of different proportions of a blue diamine monomer based on a chromogenic azadipyrromethene core, with *m*-phenyleneisophtahlamide and isophthaloyl chloride. The coloration efficiency of the chromogenic monomer is very high, and the color hue can be tuned by means of increasing or decreasing the percentage of structural units containing the dye motifs. In addition, the polyamides showed improved thermal properties compared with conventional PMIA.

### 4.9. Aromatic Polyamides with Improved Solubility

Aramids do not melt or their melting temperature (*T*_m_) or their glass transition temperatures (*T*_g_s) and softening temperatures for amorphous materials, are too high to be transformed by conventional techniques, such as injection or extrusion. Accordingly, PPTA, MPIA and ODA/PPTA are transformed upon solution into yarns to produce mainly fibers, paper and composites. However, these polymers are usually highly insoluble, characteristic that impair their application in a number of technological fields, such as in high-performance films and coatings, separation membranes and composites, to name but a few. In this section, we describe the articles mainly devoted to obtaining polyamides with enhanced solubility.

**Scheme 24:**
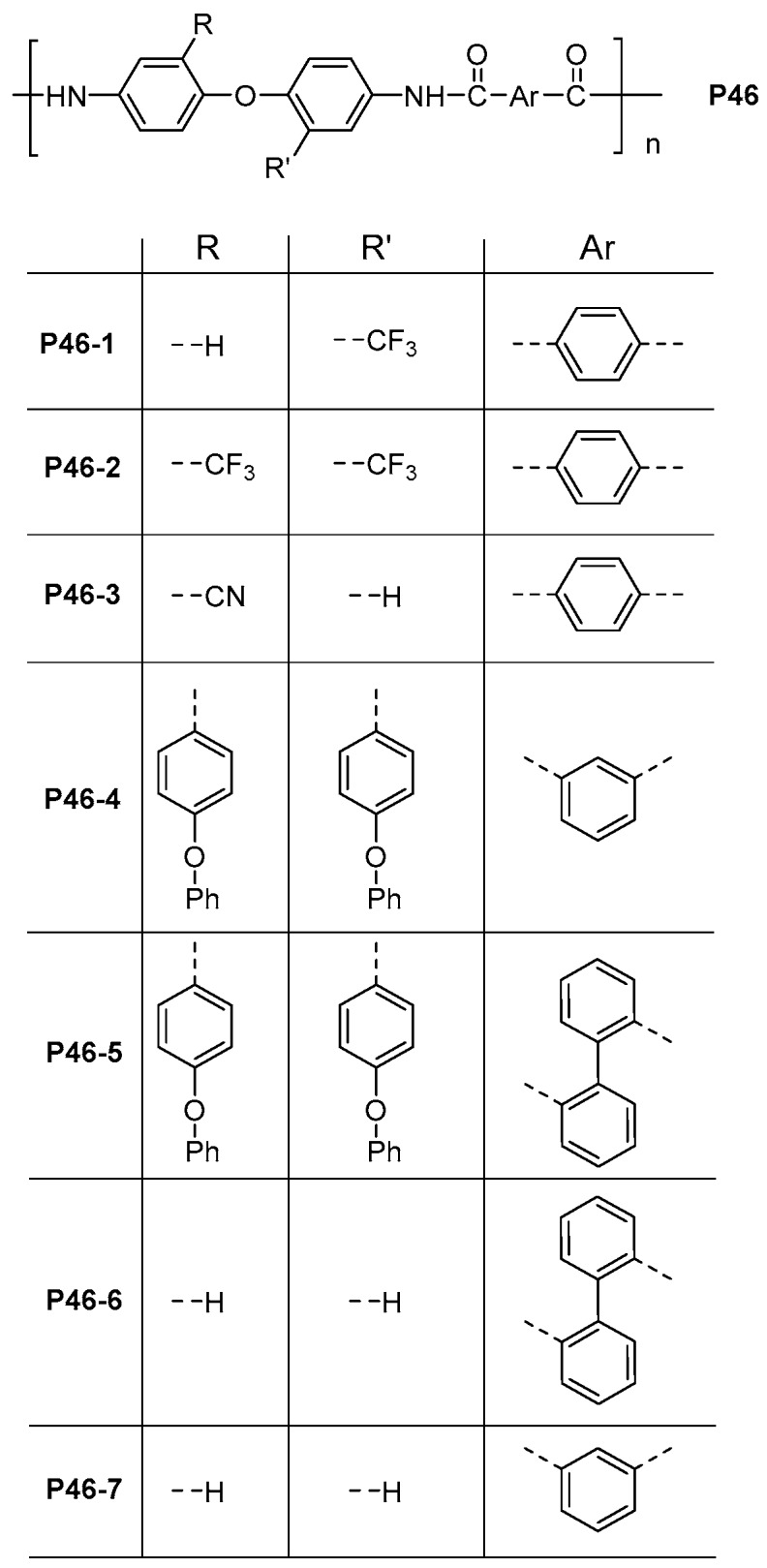
Aramids with enhanced solubility having trifluoromethyl or cyano groups in the main chain.

As it was pointed out previously, Lee et al. [93] obtained a series of *para*-linked aromatic polyamides with trifluoromethyl or cyano groups in the main chain. They were soluble in polar aprotic solvents while the aramids without these groups needed the addition of salts to be solved in aprotic solvents, even though they had flexible ether linkages, which are solubility promoters (Scheme 24, P46-1 to P46-3).

As seen before, Sadavarte et al. [28] produced polymers having lateral pentadecyl chains. The pendant aliphatic chain led to a solubility improvement. Ohsedo et al. [134] obtained water-soluble aromatic polyamide poly(3-sodium sulfo-*p*-phenylene terephthalamide) with a good thixotropic behavior, even at the critical gel concentration of the gelator (1.0 wt %).

Another approach to the improvement of the solubility and thus the processability of aromatic polyamides is the incorporation of bulky pendant groups. These bulky groups produce a separation of the chains, a concomitant weakening of hydrogen bonding, and a lowering of chain packing with a gain of free volume, which diminishes the *T*_g_. On the other hand, these bulky lateral groups also restrict molecular mobility, causing an increase in *T*_g_. So, the overall observable effect in *T*_g_ is a counterbalance of both effects.

**Scheme 25:**
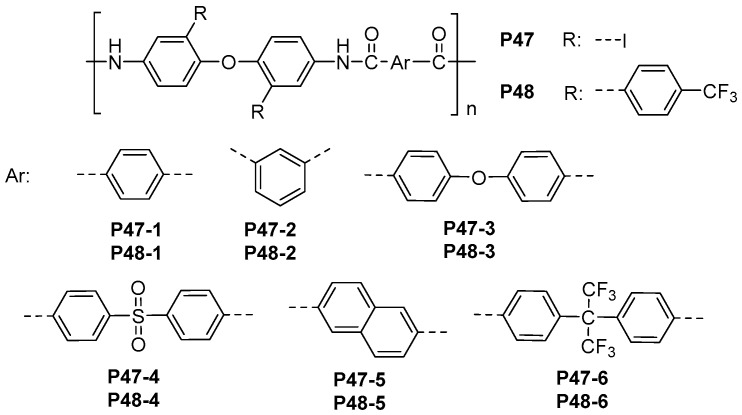
Aramids with enhance solubility containing bulky iodide and *p*-trifluoromethylphenyl groups.

In this research line, Behniafar et al. [135] prepared aromatic polyamides from the diamine 2,2′-bis(*p*-phenoxyphenyl)-4,4′-diaminodiphenyl ether and two diacids (Scheme 24, P46-4 to P46-7). The obtained polyamides exhibited excellent organo-solubility in polar solvents, *T*_g_s up to 200 °C, and 10% mass loss up to 500 °C under nitrogen. Other two diaminodiphenyl ethers with bulky iodide (Scheme 25, P47) and *p*-trifluoromethylphenyl (Scheme 25, P48) substituents were used by Chen et al. [136] with the same purpose of hindering the chain packing and increase the free volume. In addition, these polyamides showed good optical transparency in the visible light region.

**Scheme 26:**
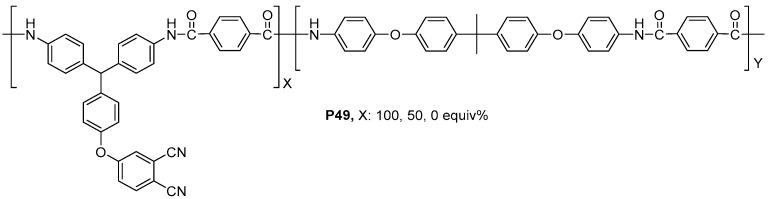
Aramids with enhanced solubility having pendant dicyanophenoxy moieties.

Soluble aromatic polyamides derived from diamine 4,4′-diamino-4″-(3,4-dicyanophenoxy)triphenylmethane and TPC were synthesized by Carja et al. [137] by introducing a flexible spacer between rigid-rod groups in order to lower the melting temperature and to improve the solubility (Scheme 26, P49). The polyamides showed good solubility in organic solvents, film forming properties, and high thermal stability, with the decomposition temperature being above 400 °C. The X-ray diffraction study showed that the presence of bulky pendant phthalonitrile groups decreased the intermolecular forces between the polymer chains causing a decrease in crystallinity. Also, *T*_g_ values depend on the rigidity of the polymer chain, since an increase in the rigidity of the polymer backbone increases the rotational energy value and thus the *T*_g_ increases. Thus, the polymer P49 that contains the highest concentration of pendant phtalonitrile groups exhibited the highest *T*_g_ (243 °C).

**Scheme 27:**
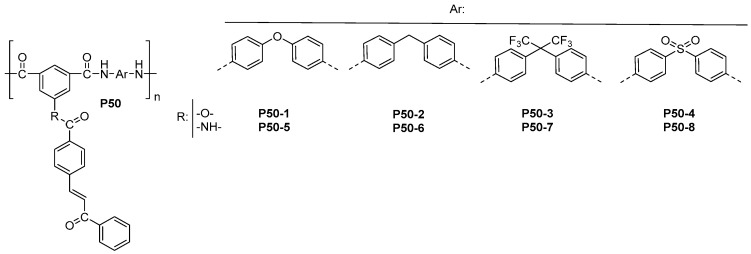
Aramids with improved solubility with pendant chalcone groups.

Another bulky and rigid side chain was introduced by Nechifor et al. [138] by anchoring a benzylideneacetophenone (chalcone) moiety through an amide or ester bridge at the fifth position of the isophthalic acid ring. A polycondensation reaction of these acids with different amines rendered polyamides with improved solubility, having an amorphous nature and good thermal stability, showing *T*_g_s 219 and 254 °C, 10% mass loss temperatures around 394–436 °C and 50% residue at 700 °C in nitrogen atmosphere (Scheme 27, P50).

**Scheme 28:**
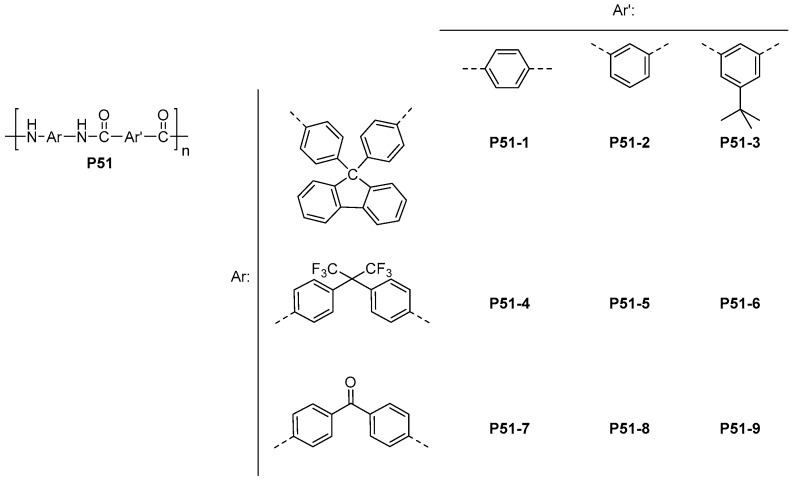
Aramids with bulky lateral groups.

Palí et al. [139] reported on the study of the influence of controlled structural modifications on physical and conformational properties of isomeric aromatic polyamides with different bulky pendant lateral groups (Scheme 28, P51). They found that there was a relationship between *T*_g_ and the conformational entropy determined by the structure, indicating that the *T*_g_ increases with increasing levels of rigidity in the polyamide. They also found a connection between the activation energy of the glass transition and the intermolecular fractional free volume (structural property which has a relationship with the conformation of chains that is a measure of the effective space between chains), determined by the presence or absence of the bulky functional groups. The smaller the interchain fractional free volume, the higher the energy required for the glass transition to take place.

The pendant loop at rigid sp^3^ carbon in cardo aramids imparts rigidity, and affects solubility and other relevant properties, such as thermal and mechanical behavior.

**Scheme 29:**
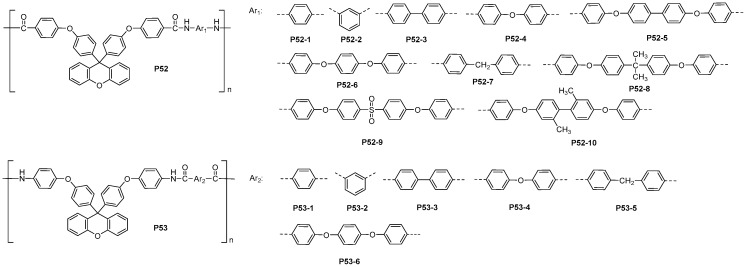
Cardo aramids.

Jian et al. [48] and Sheng et al. [59] described the synthesis of aromatic polyamides containing ether and bulky xanthene groups from a diacyl chloride monomer, 9,9-bis[4-(chloroformylphenoxy)phenyl]xanthene, or a diamine monomer, 9,9-bis[4-(4-aminophenoxy) phenyl]xanthene (Scheme 29, P52–P53). The polyamides were amorphous and soluble in polar solvents such as DMA, DMF, NMP and pyridine, showed relative high *T*_g_s (236–308 °C), decomposition temperatures at 10% mass loss from 490 to 540 °C in air, and char yields higher than 50% in nitrogen. The cast films from these polyamides were transparent and flexible with tensile strengths ranging from 82 to 109 MPa, initial moduli from 2.0 to 2.8 GPa, and elongation at break from 10 to 25%.

Fiori et al. [140] reported on a study on the solubility parameters of linear and crosslinked aramids. They found that the values of the solubility parameter through the contributions of dispersion, polar, and hydrogen bonding forces (*δ*_p_, *δ*_d_, and *δ*_h_, respectively) were equal to 8.6, 18.4, and 11.3 in linear PPTA polyamides, and on the other hand, the corresponding values of the crosslinked aromatic polyamides taken into consideration were 11.5, 16.8, and 10.2.

Hsiao et al. [141,142], prepared a series of triptycene-based poly(amide-imide)s reacting 1,4-bis(4-trimellitimidophenoxy)triptycene, 1,4-bis(4-carboxyphenoxy)triptycene, and 1,4-bis(4-aminophenoxy)triptycene with aromatic diamines or dicarboxylic acids. Comparing structurally similar poly(amide-imide)s without triptycene motifs, enhanced solubility and film-forming ability, and lower crystallization were observed.

**Scheme 30:**
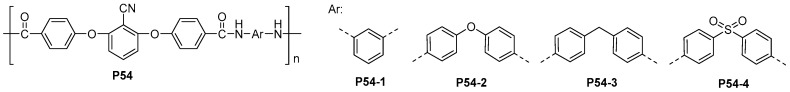
Aromatic polyamides with nitrile groups.

Pendant nitrile groups on aromatic rings in polymers are known to promote their solubility, the adhesion to many substrates through possible polar interactions with other functional groups, and can also be used as potential sites for polymer crosslinking. However, aromatic polyamides with pendant cyano groups have not been extensively reported. Yu et al. [57] described, as seen previously, the preparation and characterization of polyamides with pendant cyano groups (Scheme 30, P54) by low temperature polycondensation of aromatic diamines with 2,6-bis(4-chloroformylphenoxy)benzonitrile. The polyamides showed excellent thermal stability with 5% mass loss temperatures between 409 and 438 °C, and were soluble in polar aprotic solvents such as NMP, DMSO, DMA are swelled in solvents such as CHCl_3_, dichloromethane, tetrahydrofuran (THF) or ethylene dichloride. Films can be cast, showing good mechanical properties (tensile strength of 79–93 MPa, Young’s moduli of 1.7–2.6 GPa, elongation at break of 9–15%).

Regarding the possible crosslinking of polymers containing nitrile groups, Carja et al. described the crosslinking of the aromatic polyamides mentioned before [137] (Scheme 26, P49) through a nitrile cure reaction of polyamides around their *T*_g_. After a thermal treatment at 250 °C for 5 h, the polymers became insoluble in organic solvents and the thermal stability was increased. 

**Scheme 31:**
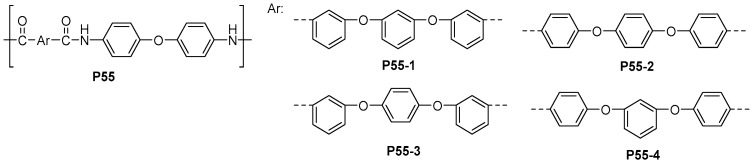
Aramids with multiple flexible ether linkages.

**Scheme 32:**
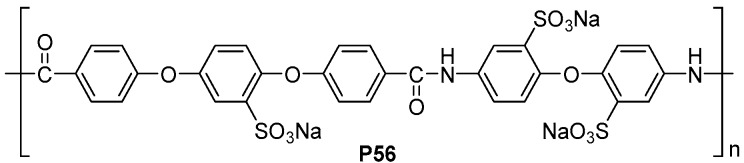
Aromatic sulfonated polyamides.

Zhao [143] described dicarboxylic acids containing flexible linkages, as a way of improving the solubility of the derived aromatic polyamide (Scheme 31, P55). The polymers showed good solubility in polar aprotic solvents, and high thermal stability with decomposition temperatures around 400 °C. Membranes out of these polyamides manifest excellent mechanical properties, with Young’s modulus of 2.5–5.5 GPa. All of the membranes were transparent except for polyamide P55-3, where crystallization was observed. The same author and coworkers presented a work [144] in which they prepared a family of multi-responsive polymers based on aromatic sulfonated polyamides (Scheme 32, P56). These smart polymers exhibited unusual and great sharp response to temperature and pH in water with the tunable low critical solution temperature. With increasing pH, the low limit critical temperature decreased, with a difference of about 60 °C when the pH is between 6.0 and 6.8, and thus polymers have potential applications as sensors.

**Scheme 33:**
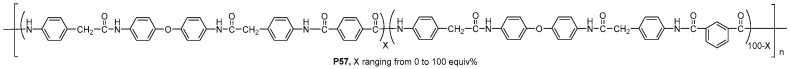
Aromatic polyamides prepared from an aromatic diamine monomer containing preformed aromatic–aliphatic amide and ether linkages.

As mentioned before, Patil et al. [49] prepared five different aromatic polyamides from an aromatic diamine monomer containing preformed aromatic–aliphatic amide and ether linkage, and different proportions of IPC or TPC (Scheme 33, P57). Polyamides were obtained in good yields with moderate to high molecular masses (η_inh_ between 0.63 and 1.35 dL/g in DMA + 4% LiCl). Partially crystalline structures were obtained, and the polyamides were soluble in polar aprotic solvents containing LiCl. The solubility of copolyamides improved due to the random distribution of constituent IPC and TPC units during polymerization. *T*_g_s were in the range of 197–204 °C, and no mass loss was observed below 336 °C.

**Scheme 34:**
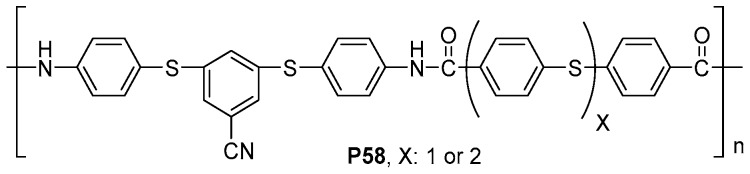
Aramids with thioether is flexible linkages.

The thioether is a flexible group and, therefore, has been introduced into polymer backbones to promote processability. According to this, Huang et al. [145] prepared, by interfacial polycondensation, aramids containing this group by reaction of 4,4′-thiodibenzoyl chloride and 4,4′-bis(4-chloroformylphenylthio)benzene with aromatic diamines containing a nitrile group (Scheme 34, P58). The η_inh_ were between 0.71 and 0.84 dL/g, they showed excellent thermal properties, with *T*_g_s and *T*_m_s in the range of 210–220 °C, and 313–315 °C, respectively. The polyamides could be melt-processed, were soluble in polar aprotic solvents, and had tensile strengths ranging from 71.3 to 79.1 MPa.

### 4.10. Designing New Materials Based on Polyamides

**Scheme 35:**
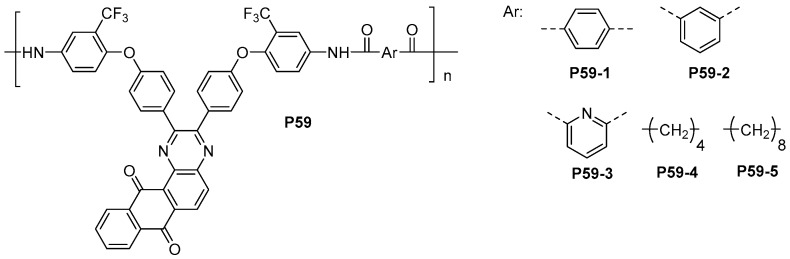
Aramids with anthraquinone-quinoxaline motifs.

As briefly seen before, polyamides can also be used to design hybrid materials in which metallic or inorganic materials are modified or functionalized introducing polyamides in their structures, such as nanoparticles, nanotubes, etc. In this research line, a few works have been published in the last years. For example, Hassan et al. [146] described the preparation of aromatic polyamides nanoparticles with lateral *m*- and *p*-acetoxybenzamide groups. The materials were fluorescent and chromogenic due to the pendant acetoxybenzamide moieties that were exploited as signaling units. Shabanian et al. [147] prepared polyamides grafted with functional carbon nanotubes. It was reported that the flammability of the aromatic polyamides was greatly reduced. As described before, Ghaemy et al. [45] prepared a series of fluorescent anthraquinone-quinoxaline containing polyamides, which were studied by cyclic voltammetry at the surface of a modified multiwalled carbon nanotubes glassy electrode. The fluorescence emission of the polymers showed maxima around 452 to 510 nm with quantum yields up to 17% (Scheme 35, P59).

Kadokawa et al. [148] analyzed the fluorescent behavior of ionically cross-linked nanoparticles produced starting with a commercial reagent, 5-aminofluorescein. The cross-linked nanoparticle was obtained by treatment with a CaCl_2_ aqueous solution. Chen et al. [149] studied a diaphragm-type flexible microactuator comprising a hyperbranched aromatic polyamide functionalized graphene filler embedded into the polyurethane dielectric elastomer matrix. Madeshnaran et al. [150], functionalized multi-walled carbon nanotubes with a hyperbranched aromatic polyamide to improve the mechanical characteristics of poly(methylmethacrylate) composites.

As previously mentioned, Yu et al. [42] prepared epoxy nanocomposites with hyperbranched aromatic polyamide grafted alumina. Thus, the glass transition temperature, thermal stability, thermal conductivity, and thermomechanical properties of nanocomposites were enhanced. The same author [151] also prepared hyperbranched aromatic polyamide grafted nano-silica particles. Yoshioka et al. [152] prepared nano-sized aromatic polyamide particles with trifluoromethyl and amino groups with diameters between 150 to 320 nm by reaction of 4,4′-diphenyldicarbonyl chloride with 2,2-bis(4-aminophenyl) hexafluoropropane and bis(3,4-diaminophenyl) ether.

**Scheme 36:**
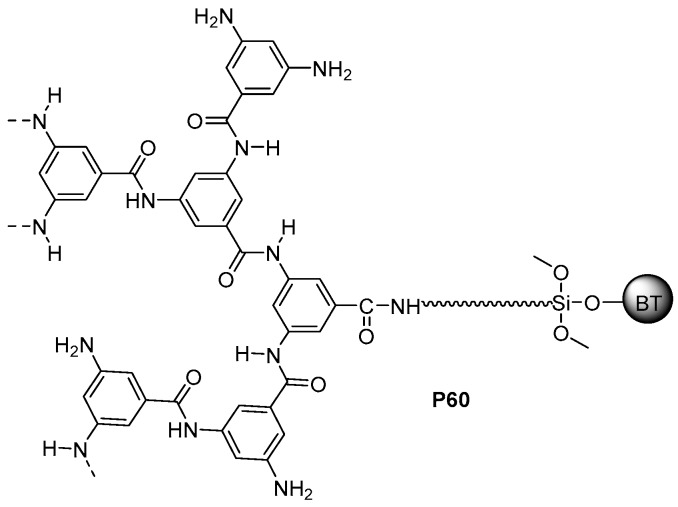
Polyamide grafted barium titanate hybrid nanofiller.

Sánchez-Sánchez et al. [153] used polyamides as precursors in the synthesis of nitrogen- and oxygen-doped ordered mesoporous carbons by applying two synthetic methods: (i) organic phase synthesis (infiltration in situ by Yamazaki reaction-based method) and (ii) solid-state thermal polymerization (liquid infiltration and thermal polymerization of the monomer into the porosity of the template). In both cases, 3-aminobenzoic acid and mesoporous silica were used as precursor and template, respectively, and the infiltration took place in one single step. After carbonization at 900 °C and removal of the template, ordered mesoporous carbons with very narrow pore size distributions and nitrogen and oxygen contents as large as ≈6 wt % and between 6.4 and 11.5 wt %, respectively. Xie et al. [154] prepared polymer nanocomposites by using core−shell structured hyperbranched aromatic polyamide grafted barium titanate hybrid nanofiller. The high intrinsic dielectric constant polymer poly(vinylidene fluoride-trifluoroethylene-chlorofluoroethylene) was used as the polymer matrix. It was found that the nanocomposite had a dielectric constant of 1485 at 1000 Hz, while the nanocomposite with untreated BaTiO_3_ exhibited a dielectric constant of 206 (Scheme 36, P60). Finally, as exposed previously, Qian et al. [43] obtained aluminum nitride nanoparticles treated with a silane coupling agent as reinforcing fillers for epoxy composites.

## 5. Selected Polyamide Structures

### 5.1. Polyamides with Heterocyclic Rings in the Main Chain

The incorporation of heterocyclic rings in the main chain or in the pendant structure of aromatic polyamides has been widely used, since they provide the polymers with excellent thermal stability and improved solubility.

**Scheme 37:**
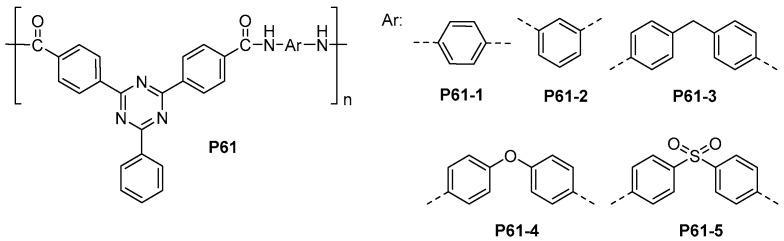
Aramids with 1,3,5-triazine moieties in the main chain.

Among these heterocyclic rings, phenyl-1,3,5-triazine has been incorporated into polyamides’ main chain to obtain a series of polymers that are soluble in NMP and DMA at room temperature and can be cast into films (Scheme 37, P61), by Yamazaki phosphorylation condensation of a diacid synthetized from 2,4,6-trichloro-1,3,5-triazine with six different aromatic diamines (see the work of Yu et al. [155]). The polyamides exhibited good mechanical properties, high *T*_g_s (311–330 °C), and excellent thermal stabilities.

**Scheme 38:**
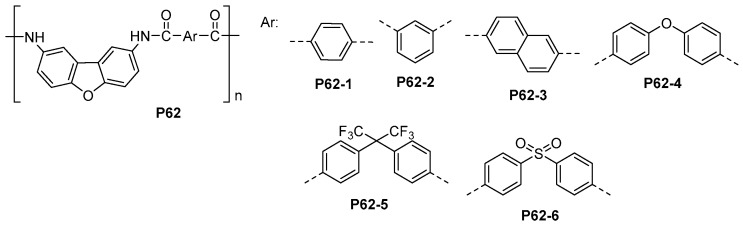
Aramids with dibenzofuran sub-structure in the main chain.

Planar dibenzofuran moieties were also incorporated into the polyamide backbone by Chen et al. [136] (Scheme 38, P62). These polyamides showed high decomposition temperatures and high *T*_g_s, resulting from their planar dibenzofuran moieties. However, in this case, solubility was not improved due to the rigidity of the planar moieties.

### 5.2. Fluorinated Polyamides

The presence of halogen atoms in the structure of aramids imparts resistance to flame, solvents, acids and bases, which are important characteristics for a number of applications. In addition, the group CF_3_ is used to increase the solubility of the polymers, and plays an important role in the electrical and dielectric performance.

**Scheme 39:**
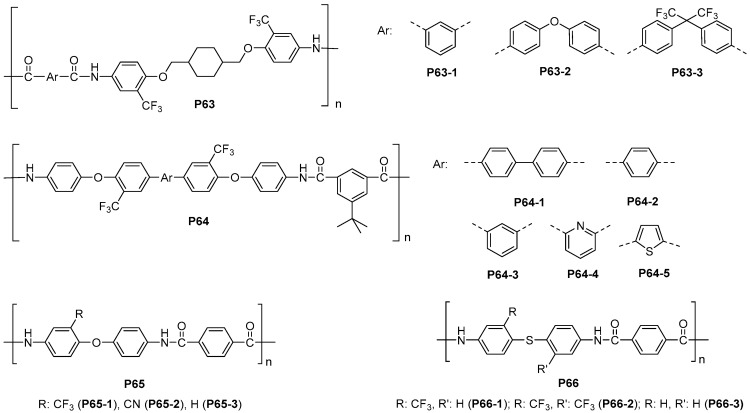
Fluorinated aramids with miscellaneous structures.

As presented previously, Li et al. [61] described de preparation of semi-aromatic polyamides derived from 1,4-bis((4-amino-2-(trifluoromethyl)phenoxy)methyl)cyclohexane with different dicarboxylic acids (Scheme 39, P63). The polymers had η_inh_ between 1.85 and 2.36 dL/g, outstanding solubility in amide-type polar aprotic solvents and even in THF and pyridine. Films prepared from these polyamides were flexible (10–12% elongation at break), transparent and tough (tensile strengths of 76.5–82.3 MPa and Young’s moduli of 1.64–1.85 GPa). They also showed low dielectric constants of 2.37–2.53 at 100 MHz. As seen before, wholly aromatic amorphous polyamides were prepared by Bera and coworkers [58] by polycondensation of different semifluorinated aromatic diamines (Scheme 39, P64) that were soluble in DMF, DMA, THF and DMSO at room temperature, having molecular masses up to 23 × 10^4^, *T*_g_ up to 261 °C and good thermal stability. Cast films out of these polyamides were clear and flexible with tensile strengths and elasticity modulus up to 88 MPa and 1.81 GPa, respectively. *Para*-linked aromatic polyamides with CF_3_ pendant groups (Scheme 39, P65–P66) were synthetized by Lee et al. [93] and compared with the same polyamides without the CF_3_ groups. The polyamides without pendant groups showed poor solubility and required LiCl to dissolve in polar aprotic solvents, while the synthesized polyamides with CF_3_ (Scheme 39, P65-1, P66-1, P66-2) groups showed enhanced solubility as well as good thermal stability. The presence of the CF_3_ groups influenced the optical properties of the polyamides, having low refractive indices (*n*), from 1.6164 to 1.6613, and low birefringence, ranging 0.0101 to 0.0305, with differences in *n*_xy_ and *n*_z_, pointing to the slight anisotropy of the aramid films.

Other examples of the role of the fluorinated groups in the improvement of the solubility and dielectric properties was evidenced by Sheng et al. [156] and Jiang et al. [44]. They synthetized fluorinated aromatic polyamides derived from the monomers 2-(4-trifluoromethylphenoxy) terephthaloyl chloride or 2-(4-trifluoromethylphenoxy)terephthalic acid with different diamines (Scheme 7). All the polyamides were soluble in polar aprotic solvents such as NMP, DMA, DMF, DMSO. Some of the polyamides, the ones with the fluorinated diamine (Scheme 7, P20), were even soluble in THF and acetone; and had η_inh_ from 0.67 to 1.16 dL/g. They show good thermal properties with 5% mass loss in the range of 430–472 °C. Tough and flexible films were cast, exhibiting good mechanical properties with tensile strengths of 71–115 MPa, elongation at break from 4.1 to 9.0%, and tensile moduli ranging from 2.26 to 3.95 GPa. Regarding the electrical isolation characteristics, the films had low dielectric constants (3.14–3.31, 1 MHz).

**Scheme 40:**
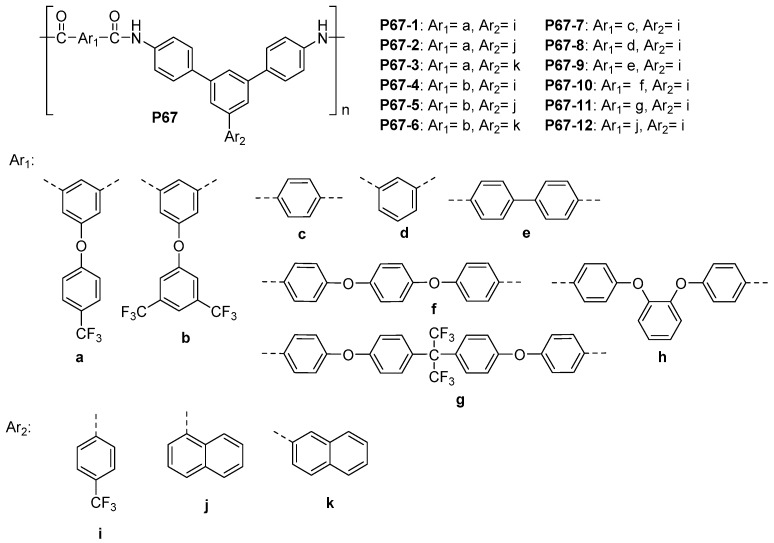
Aromatic polyamideswith trifluoromethyl moieties.

Li and coworkers [157] and Zou and coworkers [60] prepared similar aromatic polyamides (Scheme 40, P67) containing CF_3_ groups. Some polymers were even soluble in THF, had *T*_g_s between 270 and 314 °C, decomposition temperatures ranging from 442 to 516 °C (10% mass loss) and high char yields, up to 61% (800 °C) in inert atmosphere. Films were prepared by casting, having tensile strengths ranging 73–93 MPa, elongations at break of 5–14%, and initial moduli of 2.1–2.9 GPa, and acceptable electrical insulating properties.

**Scheme 41:**
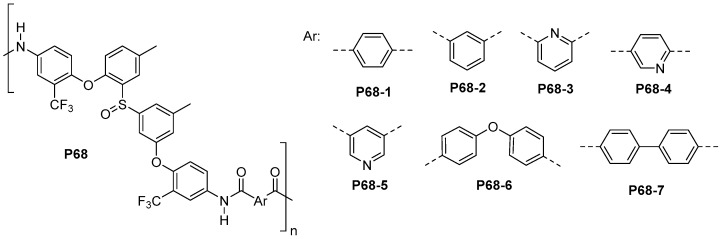
Fluorinated polyamides based on noncoplanar sulfoxide containing aromatic bis(ether amine).

As shown before, Shockravi et al. [46] reported on the synthesis of polyamides containing sulfoxide and trifluoromethyl groups from a diether-diamine (Scheme 41, P68). The resulting polymers were amorphous, had η_inh_ from 0.35 to 0.9 dL/g in DMA, showed good solubility and could be dissolved even in less polar solvents such as THF. *T*_g_s were between 160 and 220 °C, and 10% mass loss temperatures were in the range of 400–505 °C in nitrogen atmosphere. These properties, derived from the noncoplanar CF_3_ and S=O in the polymer chain, make these polyamides attractive for practical applications such as processable high-temperature-resistance polymers.

### 5.3. Miscellaneous

**Scheme 42:**
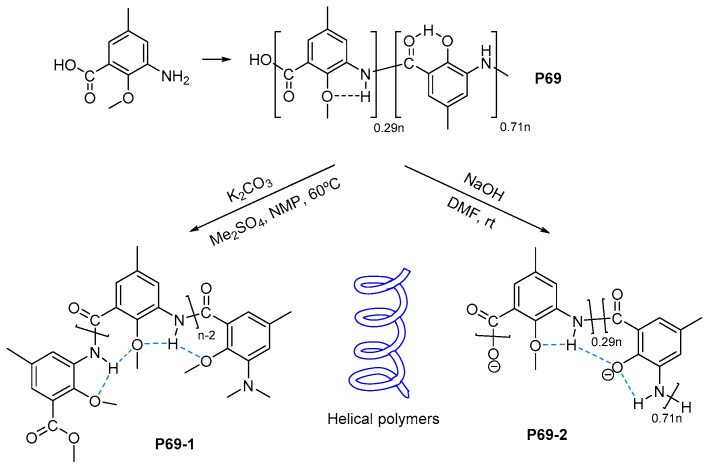
Polyamides with intramolecular hydrogen-bonding motifs that can fold into helical conformation.

Biomacromolecules such as DNA or proteins can self-assemble into well-defined structures to fulfill their functions in living systems. Trying to mimic nature, many researches have been directed into developing artificial helical polymers. Lu et al. [158] synthesized a series of polyamides with intramolecular hydrogen-bonding motifs that can fold into helical conformation (Scheme 42, P69). For that purpose, they prepared polymer P69-1 from methoxy-substituted *m*-amino benzoic acid and compared it with an analogous polymer from *m*-amino benzoic acid, with a similar structure but unable to form multiply centered intramolecular H-bonds. The polymerization was carried out following the high temperature method, and in this case LiCl was also useful to destroy intramolecular H-bonding in order to avoid cyclization during polymerization. Approximately 85% of the methoxy groups were demethylated in the resulting polymer; to address this fact, a two-step synthesis was carried out to re-methylate the polymer. Deprotonated polymer P69-2 is expected to adopt a helical structure due to similar intramolecular H-bonding pattern. The molecular mass obtained for polyamide I and II are 2430 and 2070, respectively. The chirality of helical conformation can be further induced via acid-base complexation using chiral residues and form controllable chiral helices in solution.

**Scheme 43:**
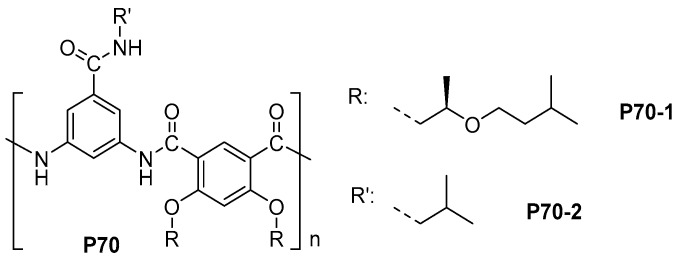
Aramids with hollow helical conformation.

In a similar way, Cao and coworkers [159] prepared folded aromatic polyamides with partially rigidified backbones by repetitive precipitation (Scheme 43, P70). Fractions of high molecular masses and low dispersity were obtained, adopting a hollow helical conformation that is stabilized by intramolecular H-bonding interactions between side chains. These polymers contain cavities with fixed diameters, and so they can lead to the preparation of porous molecular and supramolecular structures and to the development of transmembrane channels in robust nanoporous membranes.

**Scheme 44:**
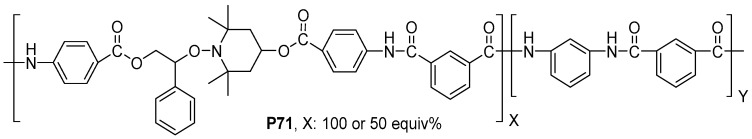
Thermally rearrangeable aromatic polyamides.

Dynamic covalent polymers have become attractive materials because their primary structures can be controlled by external stimuli, even after polymerization, and can be used in different fields. Following this idea, Aiba et al. [160] developed thermally rearrangeable aromatic polyamides and random copolyamides (Scheme 44, P71) with alkoxyamine moieties in the main chain to study the effects of this characteristic on their solution and solid-state structure. They found out that the hydrodynamic radius in solution decreased with increasing temperatures. At the same time, both the dry density and the hydrophilicity of the films decreased with increasing fabrication temperatures, caused by crossover reaction induced by temperature, and the aggregation of the hydrophobic and hydrophilic units to minimize the surface energy and suppress hydrogen bonding.

**Scheme 45:**
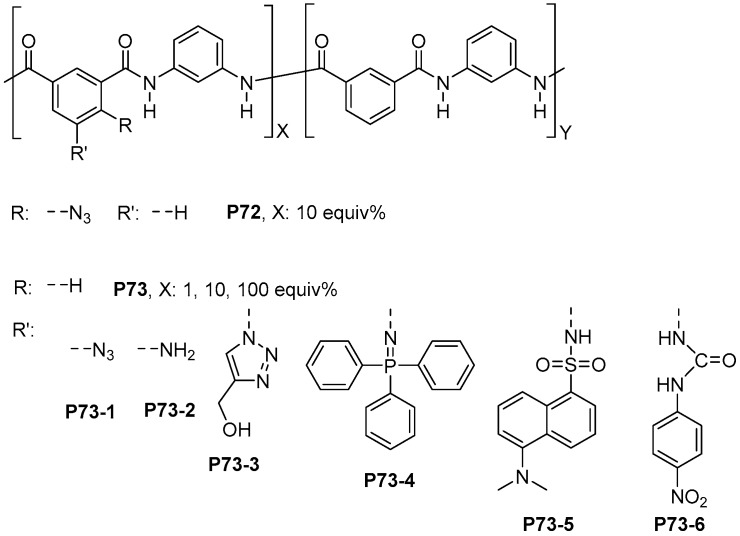
Aramids with reactive azide groups and other functional groups.

Heat can also produce crosslinking of polymeric chains as a way of improving their thermal and mechanical properties without impairing other properties, as Trigo-López [161] and coworkers demonstrated. They prepared a series of polyamides and copolyamides with reactive azide groups from *m*-phenylene diamine 5-azidoisophthaloyl chloride (Scheme 45, P72–P73). A crosslinking process was performed to cast films by heating the polyamides at 240 °C for 10 min, becoming insoluble even in strong acids, and showing an improvement in the thermal and fire resistance and mechanical performance. The crosslinking process is ascribed to the transformation of the azide groups into the highly reactive nitrene groups. Nitrene undergo different reactions causing, in this case, the crosslinking of the polyamide chains. This procedure to obtain crosslinked polyamides could be easily applied to current fiber production technologies like dry, wet or dry-jet wet spinning, to prepare advanced protection fibers, ballistic applications and electrical protection apparel, transmission belts, cut protection products, etc. The same authors also reported in a different work [162] the modification of high performance aromatic polyamides by preparing polyamides with primary amine and azide functional groups in the main chain as parent polymers. Simple and inexpensive polyamides can be prepared from the parent polymers by chemical derivatization of the reactive amino and azido groups. The potential of the parent aramids was exemplified by the preparation of polyamides with side chains containing different functionalities such as a fluorescent dansyl group P73-5, anion and cation receptors group urea and triazole with primary alcohol functional groups (P73-6, P73-3), and the iminophosphorane ligand for forthcoming synthetic processes (P73-4).

**Scheme 46:**
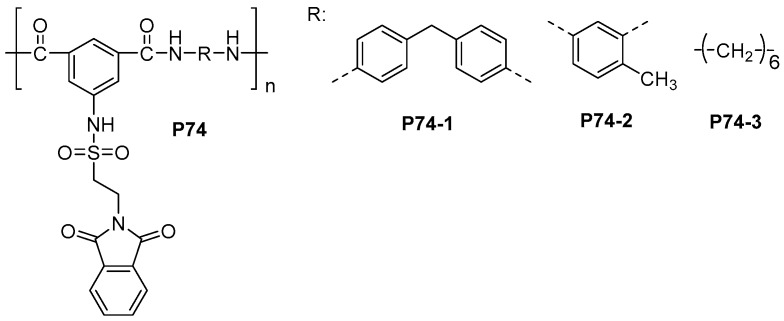
Polyamides containing phthalimidoethanesulfonamide pedant sub-structures.

The introduction of functional groups in polyamides has important effects in their properties and final applications. If the pendant group is carefully chosen, new applications can be found for aromatic polyamides. Taurine (2-aminoethanesulfonic acid) is an essential amino acid not used in protein synthesis but found to be free in simple peptides. It also has anti-epileptic activity. Karimi Zarchi et al. [163] synthetized aromatic polyamides containing 2-phthalimidoethanesulfonic acid derived from taurine as pendant group (Scheme 46, P74), which is stable at high temperature and can act as biological active unit. For that purpose, they synthesized 5-(2-phtalimidoethanesulfonamido)isophthalic acid and reacted it with aromatic and aliphatic disiisocyanates. The hydrogenase activities of treated soils with the polyamides were studied, showing that it was 2–3 times higher, indicating that the activity of the microbial communities was enhanced by the presence of polymers. This means that the polyamides are biodegradable under soil burial environments and that they are not toxic for soil microorganisms. Rafiee et al. [53] also prepared biodegradable polymers previously mentioned (Scheme 9, P24).

**Scheme 47:**
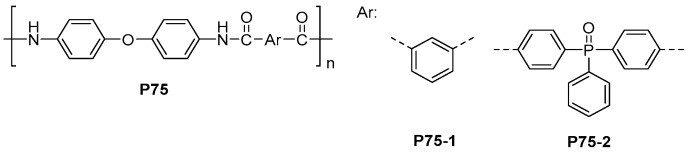
Aromatic polyamides with phenylphosphine oxide group.

Other group introduced in aromatic polyamides is phenylphosphine oxide. Su and coworkers [164] prepared a polyamide containing this group (Scheme 47, P75). The results showed that this group protects the aramid film from erosion by atomic oxygen.

**Scheme 48:**
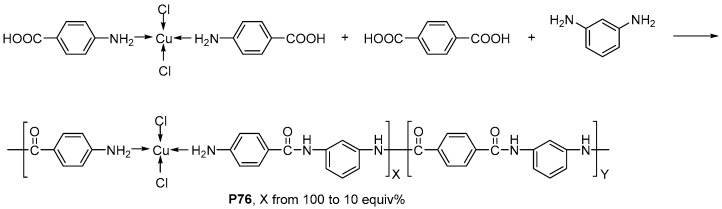
Polyamides with coordination metal complex units.

The vast majority of the polyamides reported in the literature are non-metal organic chemicals and relatively little research has been made regarding metal containing aromatic polyamides. However, Zhang et al. [165] described the possibility of the production of organosoluble polyamides with coordination metal complex units in the main chain (Scheme 48, P76). They used copper(II) coordination complex as a monomer and reacted it with *m*-phenylenediamine to obtain a low molecular mass polyamide (η_inh_ = 0.20 dL/g). A higher molecular mass copolyamide was obtained using different proportions of *p*-phthalic acid as a comonomer. Ultraviolet absorption properties and solubility were enhanced significantly by the incorporation of Cu(II) ions in the polymer chains.

## 6. Conclusions and Outlook

The demand of commercial aramid fibers derived from the spun of MPDI, ODA/PPTA and PPTA, is strong, e.g., 100–120 thousands of tons expected for PPTA in 2020, with an expected annual growth range of 7–9% [6]. This fact is mainly related to the application of the fibers in protective clothing, advanced composites and heat and fire resistant insulation paper. The advances in aramid research summarized in this review point to the opportunity to expand the application field of the aromatic polyamides by increasing the outstanding thermal and mechanical resistance of the materials, or by using this resistance in new fields related to chemical sensing, advanced extraction techniques, or exploitation of many characteristics, such as optical, electrical or magnetic properties.

## Figures and Tables

**Table 1:** Structure, aramid type, brand names and company of commercial aramids.

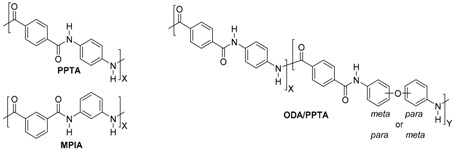				
Aramid type			Brand names	Company
PPTA	MPIA	ODA/PPTA		
X			Kevlar^®^	DuPont, Wilmington, DE, United States
	X		Nomex^®^	
X			Twaron^®^	Teijin, Arnhem, the Netherlands
	X		Teijinconex^®^	
		X	Technora^®^	
X			Heracron^®^	Kolon Industries, Seoul, Korea
X			Alkex^®^	Hyosung, Seoul, Korea
	X		*Meta* Aramid Yarn & Thread^®^	SRO Group, Heatherbrae, New South Wales, Australia
X			Taparan^®^ *Para*-aramid	Yantai Tayho Advanced Materials Co., Ltd, Yantai, Shandong, China
	X		Newstar^®^ *Meta*-Aramid	
	X		Arawin^®^	Toray Chemical Korea Inc., Seoul, Korea
	X		Metaone	Huvis, Seoul, Korea

**Table 2:** Summary of the mechanical properties.

Reference	Tensile strength (MPa)	Young’s moduli (GPa)	Elongation at break (%)
[44]	71–115	2.7–3.2	6–9
[55]	63.9–81.6	–	7.2–11.4
[56]	79–93	1.7–2.6	9–15
[57]	Up to 88	Up to 1.81	Up to 25
[58]	82–106	2.0–2.8	10–25
[59]	86–109	2.15–2.63	13–22
[60]	72.5–87.3	2.35–2.87	5.3–9.5
[61]	Up to 131	Up to 3.5	Up to 4.5

**Table 3:** Summary electroactive sub-groups in electrochromic aramids.

Reference	Main electroactive group	Auxiliary EDG or EWG	Comments
[41]	triphenylamine, TPA	--	hyperbranched
[115]		--	
[121]		3,6-dimethoxycarbazole	
[112,122]		3,6-di-tert-butylcarbazole	
[96,101,103]		methyl, trifluoromethyl	
[104]		4-*tert*-butyl	
[99,116,117,118]		2,4-dimethoxy	
[102]		adamantylphenoxy	
[105]		--	ether-linked bis(triphenylamine)
[106]		piperidinyl	
[108]		morpholinyl	
[97]	TPA (pendant)	--	
[107,109,113]	TPA or carbazole		electrompolymerization
[110,124,125]	diphenylpyrenylamine		
[98]	carbazole	methoxy	
[114]	carbazole (pendant)		
[119,120,123]	*N*,*N*,*N*′,*N*′-tetraphenyl-1,4-phenylenediamine, TPPA	*tert*-butyl	
[111]		methoxy	
[100]	bis(diphenylamino)naphthalene	metoxy	
[126]	diphenylfluorenylamine	methyl	
[127]	aniline pentamer/azo group		

## Glossary

DMA	*N*,*N*-dimethylacetamide
DMF	*N*,*N*-dimethylformamide
DMSO	dimethyl sulfoxide
DSC	differential scanning calorimetry
EDG	electron-donating group
EWG	electron-withdrawing group
η_inh_	inherent viscosity
IPC	isophthaloyl dichloride
*M*_n_, *M*_w_	number-average and weight-average molecular masses
MPD	*m*-phenylenediamine
NMP	*N*-methyl-2-pyrrolidone
ODA	3,4-diaminodiphenyl ether
ODA/PPTA	3,4-diaminodiphenyl ether/poly(*p*-phenylene terephthalamide)
MPIA	poly(*m*-phenylene isophthalamide)
POSS	polyhedral oligomeric silsesquioxane
PPD	*p*-phenylenediamine
PPTA	poly(*p*-phenylene terephthalamide)
*T*_g_	glass-transition temperature
*T*_m_	melting temperature
TEG	tri(ethylene glycol) monomethyl ether
THF	tetrahydrofuran
TPA	triphenylamine
TPPA	*N*,*N*,*N*′,*N*′-tetraphenyl-1,4-phenylenediamine
TPC	terephthaloyl dichloride
